# Evaluation and Application of a PET Tracer in Preclinical and Phase 1 Studies to Determine the Brain Biodistribution of Minzasolmin (UCB0599)

**DOI:** 10.1007/s11307-023-01878-7

**Published:** 2023-12-18

**Authors:** Joël Mercier, Massimo Bani, Anny-Odile Colson, Massimiliano Germani, Marianna Lalla, Christophe Plisson, Mickael Huiban, Graham Searle, François-Xavier Mathy, Richard Nicholl, Christian Otoul, Johan Willem Smit, Vanja van Asch, Michel Wagneur, Ralph Paul Maguire

**Affiliations:** 1 UCB Pharma, Braine L’Alleud, Belgium; 2 Present Address: OxSonics, Oxford, UK; 3 Invicro, London, UK; 4 UCB Pharma, Slough, UK; 5 Present Address: Curare Consulting, Hamburg, Germany; 6 Auximines Clinical Solutions, Zemst, Belgium

**Keywords:** Minzasolmin, Parkinson’s disease, Alpha-synuclein, PET-imaging, Brain distribution, PET tracers, Phase 1 study

## Abstract

**Purpose:**

Minzasolmin (UCB0599) is an orally administered, small molecule inhibitor of ASYN misfolding in development as a potential disease-modifying therapy for Parkinson’s disease. Here we describe the preclinical development of a radiolabeled tracer and results from a phase 1 study using the tracer to investigate the brain distribution of minzasolmin.

**Procedures:**

In the preclinical study, two radiolabeling positions were investigated on the *S*-enantiomer of minzasolmin (UCB2713): [^11^C]methylamine UCB2713 ([^11^C-*N*-CH_3_]UCB2713) and [^11^C]carbonyl UCB2713 ([^11^C-CO]UCB2713). Male C57 black 6 mice (*N* = 10) received intravenous [^11^C-*N*-CH_3_]UCB2713; brain homogenates were assessed for radioactivity and plasma samples analyzed by high-performance liquid chromatography. Positron emission tomography-computed tomography (PET-CT) was used to image brains in a subset of mice (*n* = 3). In the open-label, phase 1 study, healthy volunteers were scanned twice with PET-CT following injection with [^11^C]minzasolmin radiotracer (≤ 10 µg), first without, then with oral dosing with non-radiolabeled minzasolmin 360 mg. Primary objective: to determine biodistribution of minzasolmin in the human brain; secondary objectives included minzasolmin safety/tolerability.

**Results:**

Preclinical data supported the use of [^11^C]minzasolmin in clinical studies. In the phase 1 study, PET data showed substantial drug signal in the brain of healthy volunteers (*N* = *4*). The mean estimated whole brain total distribution volume (V_T_) at equilibrium across all regions of interest was 0.512 mL/cm^3^, no difference in V_T_ was observed following administration of minzasolmin 360 mg. Treatment-emergent adverse events (TEAEs) were reported by 75% (*n* = 3) of participants. No drug-related TEAEs, deaths, serious adverse events, or discontinuations were reported.

**Conclusion:**

Following positive preclinical results with the N-methyl labeled PET tracer, [^11^C]minzasolmin was used in the phase 1 study, which demonstrated that minzasolmin readily crossed the blood–brain barrier and was well distributed throughout the brain. Safety and pharmacokinetic findings were consistent with previous early-phase studies (such as UP0077, NCT04875962).

**Supplementary Information:**

The online version contains supplementary material available at 10.1007/s11307-023-01878-7.

## Introduction

Parkinson’s disease (PD) is a progressive, neurodegenerative disease, resulting in motor and non-motor symptoms [1]. Neuropathological characteristics of PD include loss of dopaminergic neurons in the substantia nigra and formation of Lewy bodies comprising alpha-synuclein (ASYN) [2]. ASYN misfolding results in aggregates of oligomeric, beta-sheet rich structures that form fibrils and Lewy body pathology within neuronal cells. This misfolding is an early step in a pathological cascade which leads to neuronal cell death and the spread of aggregated ASYN protein from neuron to neuron [3–5]. ASYN aggregation plays a central role in the development of PD, therefore understanding the biochemistry of ASYN is key to developing treatments for the disease [4, 6]. There are currently no treatments that slow/halt the progression of PD [1]; current treatments provide symptomatic relief but become less effective at managing symptoms in later stages of PD [7].

Minzasolmin is an orally administered, small molecule that acts on the early steps of the ASYN cascade, specifically inhibiting ASYN misfolding [8, 9] and is under clinical investigation for the potential to reduce PD pathology and slow progression of disease [8]. In a mouse model of PD, once-daily administration of minzasolmin over 3 months was well-tolerated and reduced disease-related ASYN pathology, neuroinflammation, and motor impairments [8].

Minzasolmin acts at the lipid membrane [9] and has previously been shown to penetrate the blood–brain barrier and distribute into the cerebrospinal fluid (CSF). The ratio of CSF concentration to free plasma concentration (Kp,uu) ranged between 0.6–0.9 across a range of doses [10] and the concentration of minzasolmin that can be achieved in the brain parenchyma and on cell membranes may be important to its mode of action. It has also been shown that transport of minzasolmin across the blood–brain barrier is not affected by efflux pumps; when tested *in vitro* in the Caco-2 cell system, minzasolmin showed a high permeability without any active transport (efflux ratio [ER] ≤ 2) [10].

**Fig. 1:**
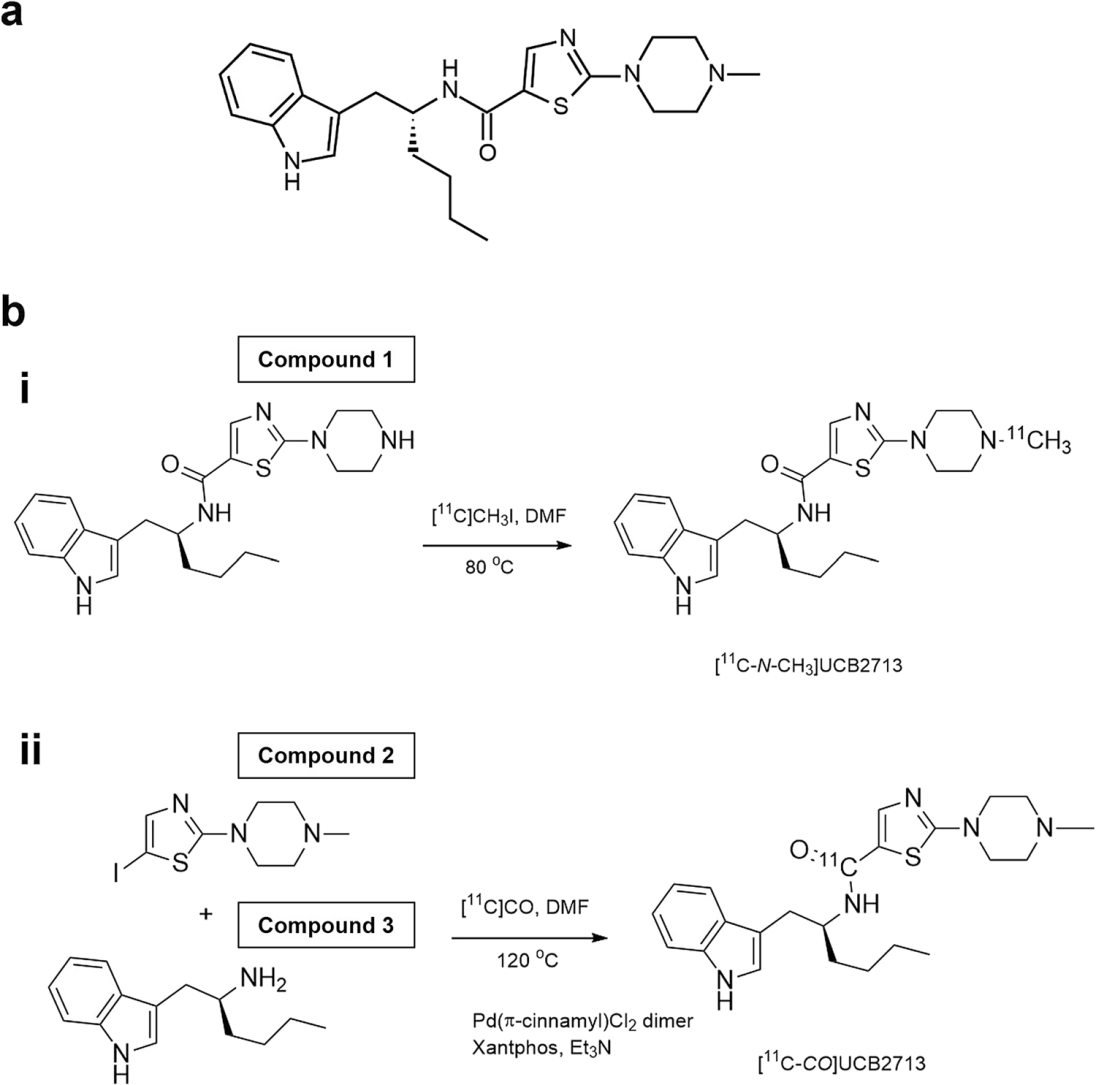
**a** Chemical structure of minzasolmin. UPAC Nomenclature: N-[(1R)-1-(1H-indol-3-ylmethyl)pentyl]-2-(4-methylpiperazin-1-yl)thiazole-5-carboxamide. Molecular weight: 425.6, **b** Radiosynthesis of [^11^C-*N*-CH_3_]UCB2713 on the *N*-piperazine site (i) and [^11^C-CO]UCB2713 by [^11^C]aminocarbonylation (ii). *Compound 1* N-[(1R)-1-(1H-indol-3-ylmethyl)pentyl]-2-piperazin-1-yl-thiazole-5-carboxamide*, Compound 2* 5-iodo-2-(4-methylpiperazin-1-yl)thiazole*, Compound 3* (2S)-1-(1H-indol-3-yl)hexan-2-amine, *[*^*11*^*C-CO]UCB2713* [^11^C]carbonyl UCB2713, *[*^*11*^*C-N-CH*_*3*_*]UCB2713* [^11^C]methylamine UCB2713, *DMF* dimethylformamide, *Et*_*3*_*N* triethylamine.

Minzasolmin (Fig. 1a) was purified from NPT200-11, a racemic mixture that has been purified to single enantiomers: the *R*-enantiomer, minzasolmin; and the *S*-enantiomer, UCB2713. Previous studies demonstrated that interactions between membrane-bound ASYN and the two enantiomers are identical [9]. The preclinical radiolabeling study in mice was completed with UCB2713 to determine feasibility of radiolabeling the enantiomers. After sufficient radiochemical yield of UCB2713 was observed with one of two radiolabeling positions, the clinical study was then completed with minzasolmin. UCB2713 was used in the preclinical radiolabeling study in mice, as it had a higher rate of metabolism compared with the *R*-enantiomer, minzasolmin. Given that the point of interest of this study was to observe metabolism in the brain, there would be a greater chance of observing metabolites in the brain by using the enantiomer with a higher rate of metabolism from the outset.

Determination of pharmacokinetics and distribution of investigational medicines is important in their clinical development, with particular focus on their ability to reach target organs. Distribution of agents in the brain can be assessed non-invasively via positron emission tomography (PET) and radiolabeling the investigational medicine can allow radiotracer quantitation of the concentration in the brain; a good regulatory framework is available for such studies in humans [11–13]. PET is a highly sensitive technique and is unique in allowing non-invasive detection of low concentrations of radiolabeled compounds in tissues that are difficult to reach, such as the brain (providing the radiolabeled tissue is distinguishable from the surrounding signal) [11, 13].

We describe results of two studies; an evaluation study of [^11^C]methylamine UCB2713 ([^11^C-*N*-CH_3_]UCB2713; hereafter, [^11^C]UCB2713) *in vivo*/*ex vivo* in mice, followed by a phase 1 clinical study (TM0017), assessing the brain penetration and biodistribution of minzasolmin using PET imaging. We compare scans performed after a radiotracer dose (≤ 10 µg) of [^11^C]minzasolmin alone and after radiotracer injection following a 360 mg oral dose of non-radiolabeled minzasolmin. Safety and tolerability of orally administered minzasolmin were also assessed in the phase 1 study.

## Methods

### [^11^C]UCB2713 *in vivo*/*ex vivo* Evaluation

#### Radiosynthesis of UCB2713

The objective of the preclinical study in mice was to evaluate the feasibility of radiolabeling UCB2713 at two different positions, to assess which compound would be most suitable for a future phase 1 study to evaluate the brain biodistribution of minzasolmin. To assess the brain biodistribution of minzasolmin in humans, the radiolabeling position should ideally be metabolically stable, for example, the [^11^C]carbonyl position.

In this preclinical study, two radiolabeling positions were investigated, [^11^C-*N*-CH_3_]UCB2713 and [^11^C]carbonyl UCB2713 ([^11^C-CO]UCB2713). The radiolabeling of [^11^C-*N*-CH_3_]UCB2713 was performed by *N*-methylation of the corresponding desmethyl precursor with [^11^C]methyl iodide and radiolabeling of [^11^C-CO]UCB2713 was performed by [^11^C]aminocarbonylation of the iodo precursor and the amino precursor with [^11^C]carbon monoxide in the presence of Pd(π-cinnamyl) chloride dimer and Xantphos (4,5-bis(diphenylphosphino)-9,9-dimethylxanthene) (Fig. 1b). For additional information on the precursor molecules and radiolabeling methods, see the electronic supplement. A high-performance liquid chromatography (HPLC) metabolite analysis method was developed to quantify the ratio of [^11^C-*N*-CH_3_]UCB2713 and other unidentified metabolites in plasma and brain homogenate samples. Relative quantification was used to provide the ratio of parent to metabolite in these samples.

#### Experimental Procedures

Preclinical experiments were conducted on male C57 black 6 mice (*N* = 10). *In vivo* experiments were performed on 3 mice and included a PET scan of the brain; *ex vivo* experiments were performed on all 10 mice and included samples of brain homogenate.

All experiments were performed in accordance with the United Kingdom (UK) Animals (Scientific Procedures) Act 1986 and the transposed European Union (EU) directive 2010/63/EU. Procedures used in this study were approved by the Animal Ethical Review Committee of Imperial College London.

For more information on the experimental procedures (administration of radioligand, collection of samples, brain sample processing, preparation of sample for analysis, plasma sample analysis, HPLC procedure and data analysis, PET-computed tomography (CT) scanning and image reconstruction, and PET-CT image data analysis) see the electronic supplement, including Supplemental Figs. 1 and 2.

### Phase 1 PET Study in Healthy Male Participants (TM0017)

#### Study Design and Eligibility

**Fig. 2:**
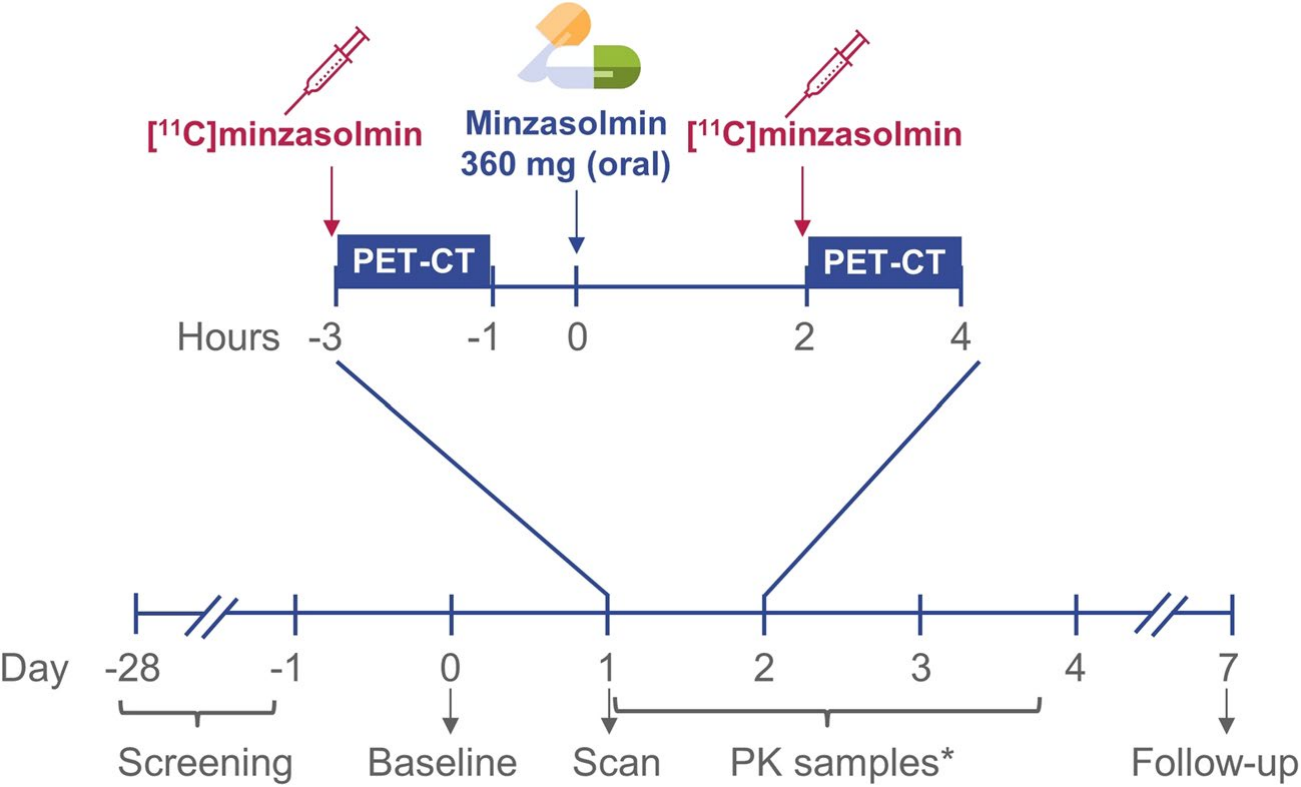
Study design figure for TM0017. *PK samples were taken at 0 h (up to 5 min before dosing), and 0.25 h, 0.5 h, 1 h, 1.5 h, 2 h, 3 h, 4 h, 6 h, 8 h, 10 h, 12 h, 15 h, 24 h, 36 h, and 48 h after 360 mg oral minzasolmin. *h* hours, *min* minutes, *PET-CT* positron emission tomography-computed tomography, *PK* pharmacokinetics.

A phase 1, single-center, open-label study was designed to determine the brain biodistribution of tracer [^11^C]minzasolmin with PET imaging in healthy male participants; safety and tolerability of minzasolmin was also assessed. Participants were scanned twice with PET-CT, radiotracer injections of [^11^C]minzasolmin (≤ 10 µg) were administered ahead of each scan and following the first scan a 360 mg oral dose of minzasolmin was administered 2–4 h before the second scan (Fig. 2). A sample size of 2–10 participants was considered sufficient to meet the objectives, whilst limiting radiation exposure.

All participants in the study were healthy, non-smokers, male, ≥ 25 and ≤ 55 years of age, with a body weight of ≥ 50 kg, body mass index of ≥ 18.0 or ≤ 30.0 kg/m^2^, with adequate collateral flow to the radial and ulnar arteries in both hands, as determined by an Allen’s test. Key exclusion criteria for this study included a history of neurological disease or an abnormal brain structure, as assessed by magnetic resonance (MR) imaging.

This study was conducted under the auspices of an Independent Ethics Committee and in accordance with the current version of the applicable regulatory and International Council for Harmonization Good Clinical Practice requirements, the ethical principles that have their origin from the Declaration of Helsinki, and the local laws of the countries involved.

#### Objectives and Variables

The primary objective was to determine the biodistribution of minzasolmin in the human brain. Secondary objectives included evaluating the safety and tolerability of minzasolmin, determining the ratio between the concentration of minzasolmin in the brain and that in plasma, and determining the relationship between brain biodistribution and administered dose of minzasolmin.

#### The Generation of Radiolabeled [^11^C]minzasolmin

[^11^C]minzasolmin was prepared as a sterile solution and was infused via IV bolus at a maximum dose of 300 MBq (≤ 10 µg) during each PET-CT scan. [^11^C]minzasolmin was prepared under Good Manufacturing Practice conditions, in accordance with local Chemistry, Manufacturing, and Control procedures and quality specifications described in the local radiopharmacy manual.

#### PET-CT Imaging Procedures and Scanning

The preparation of radiopharmaceuticals and execution of PET-CT scans was performed by radiochemists, physicians, and technologists of Invicro (London, UK). Following local anesthesia, a catheter was inserted into the radial artery by an experienced physician; two venous catheters were also inserted. A maximum of 300 MBq of [^11^C]minzasolmin was administered by qualified personnel after participants had been positioned in the PET scanner. Upon administration, dynamic scanning started immediately and continued for 90 min. Each participant underwent two PET-CT scans; approximately 2 h before the second scan the participants took a single oral dose of minzasolmin 360 mg.

During PET-CT scanning, continuous and discrete blood samples were collected with sufficient frequency to allow accurate determination of the time course of arterial whole blood, plasma, and radio-metabolites of [^11^C]minzasolmin. Arterial input function data were acquired and processed for all PET scans.

#### PET-CT Image Analysis

The outcome parameter for the assessment of minzasolmin availability was the estimated whole brain total distribution volume (V_T_) at equilibrium, unless significant heterogeneity between brain regions was observed. Anatomical regions of interest (ROI) were defined via nonlinear deformation of a template MR image and associated neuroanatomical atlas, to match individual participants’ MR images. These ROI included, but were not limited to: parietal lobe, occipital lobe, frontal lobe, temporal lobe, striatum, cerebellum, thalamus, and the brain stem. Participants’ PET images were registered to their MR images, enabling the individualized ROI to be applied to the dynamic emission data to generate regional time activity curves (TACs). For additional information on the generation of regional TACs, see the electronic supplement.

#### Safety Measurements

Adverse events (AEs) experienced by participants during this study and their medical history were coded using Version 20.1 of the Medical Dictionary for Regulatory Activities [14]. Medications were coded according to the September 2017 version of the World Health Organization Drug Dictionary [15]. A treatment-emergent adverse event (TEAE) was defined as any AE with a start date at the time of or after the first administration of [^11^C]minzasolmin. Where dates were missing or partially missing, AEs were assumed to be treatment-emergent, unless there was clear evidence to suggest otherwise.

Other safety variables recorded include changes in 12-lead electrocardiogram (ECG) assessment and changes in vital signs (heart rate, systolic blood pressure, diastolic blood pressure, respiratory rate, and body temperature). Vital signs were measured in supine position following 5 min of rest and clinically significant abnormalities (according to the investigator) were recorded as an AE. All ECG recordings were performed following 5 min of rest in the supine position, in triplicate, with ≥ 1 min between each recording. If any clinically significant abnormalities occurred, an ECG data specialist was to review the recordings.

### Statistical Methods

All statistical analyses were performed using SAS^®^ version 9.4 (SAS Institute, Cary, NC, USA). Descriptive statistics were produced if there were three or more data points for a given dose level at a given time.

## Results

### Radiosynthesis of the UCB2713 PET Tracers

[^11^C-*N*-CH_3_]UCB2713 was successfully radiolabeled with carbon-11, which enabled its use for *in vivo* evaluation in mice. Total synthesis time was approximately 35 min, including purification and formulation counted from the end of bombardment. The reaction purification carried out by semi-preparative HPLC resulted in [^11^C-*N*-CH_3_]UCB2713 of high chemical and radiochemical purity. The radiochemical purity, determined by analytical HPLC with radio-detection, was measured as 100% given that no other radioactive entity could be detected. The mean (SD) molar activity of [^11^C-*N*-CH_3_]UCB2713 was 28.1 (± 18.0, n = 5) GBq/µmol at the end of synthesis. The mean (SD) concentration of unlabeled tracer was 10.7 (± 9.8, n = 5) µg/mL. The mean (SD) radiochemical yield was 1462.2 (± 611.2, n = 5) MBq at the end of synthesis, corresponding to a 18.7% decay-corrected radiochemical yield, based on estimated [^11^C]CO_2_ produced.

[^11^C-CO]UCB2713 was also successfully labeled via aminocarbonylation using [^11^C]carbon monoxide, however, the radiochemical yield obtained with this radiosynthetic route was significantly lower than that of the labeling in the methyl position. The total synthesis time was also approximately 35 min, including purification and formulation counted from the end of bombardment. The reaction purification carried out by semi-preparative HPLC resulted in [^11^C-CO]UCB2713 in high chemical and radiochemical purity. The radiochemical purity, determined by analytical HPLC with radio-detection, was measured as 100% given that no other radioactive entity could be detected. The radiochemical yield was low with a mean (SD) of 102.3 (± 87.4, n = 4) MBq at the end of synthesis, corresponding to a 0.7% decay-corrected radiochemical yield based on estimated [^11^C]CO_2_ produced. The mean (SD) molar activity of [^11^C-CO]UCB2713 was  > 32.0 (± 34.5, n = 4) GBq/µmol at the end of synthesis and the mean concentration of unlabeled UCB2713 was less than 6.475 µg/mL.

### [^11^C]UCB2713 *in vivo/ex vivo* Evaluation

During the *in vivo*/*ex vivo* evaluation of [^11^C]UCB2713, the feasibility of radiolabeling UCB2713 at two different positions was assessed. Results indicated that [^11^C-*N*-CH_3_]UCB2713 showed higher decay-corrected radiochemical yield compared with [^11^C-CO]UCB2713, hence the [^11^C]UCB2713 referred to in this section is [^11^C-*N*-CH_3_]UCB2713.

### Mouse Brain and Plasma Extraction Efficiency

For the 10 mice, extraction efficiency was determined at 40, 60, and 90 min post-administration of [^11^C]UCB2713 for brain and plasma samples (Table 1). The plasma and brain extraction efficiency were satisfactory, with mean values (SD) of 77.4% (± 11.0%, n = 10) and 84.1% (± 4.0%, n = 10), respectively, using UCB2713 as a reference standard. The first two brain and plasma samples were extracted with acetonitrile at a ratio of 1:1 (sample:acetonitrile); results of the study showed that increased acetonitrile was associated with increased extraction efficiency. Therefore, all subsequent samples were extracted with acetonitrile at a ratio of 1:2 (sample:acetonitrile).

**Table 1:** Metabolite analysis of [^11^C-*N*-CH_3_]UCB2713 in mouse brain and plasma

% Parent				HPLC recovery		Extraction efficiency	
Subject	Time (min)	Plasma	Brain	Plasma	Brain	Plasma	Brain
Mouse 2	60	19.5	40.9	92.7	79.5	75.4^a^	82.3^a^
Mouse 3	90	26.0	29.7	58.7	104.4	59.9^a^	76.8^a^
Mouse 4	40	29.3	56.0	94.3	93.6	82.5	87.9
Mouse 5	40	30.8	58.2	97.3	105.0	83.4	85.3
Mouse 6	60	24.6	50.3	90.1	87.3	78.4	85.7
Mouse 7	90	17.7	22.1	86.6	103.6	51.7	77.6
Mouse 8	90	9.7	34.7	90.6	87.0	73.4	78.1
Mouse 9	40	34.3	62.0	ND^b^	94.6	83.1	86.2
Mouse 10	60	30.6	52.6	81.6	85.6	84.1	87.2
Mouse 11	40	27.0	61.1	93.7	100.0	82.9	84.9
Mean				90.9		77.4	84.1
SD				10.6^c^		11.0	4.0

^a^ Extraction 1:1 homogenate or plasma: acetonitrile; ^b^ HPLC recovery was not determined due to lack of plasma available; ^c^Mean and SD are for both brain and plasma

*[*^11^C-*N*-CH_3_*]UCB2713* [^11^C]methylamine UCB2713*, HPLC* high-performance liquid chromatography, *min* minute, *ND* not determined, *SD* standard deviation

### HPLC Recovery

The recovery of the radioactivity injected onto the HPLC column was determined for each mouse brain and plasma sample. The mean (SD) HPLC recovery was 90.9% (± 10.6%, n = 10; Table 1), relatively high, indicating that there were no major apolar metabolites retained on the HPLC column.

### PET imaging and *ex vivo* experiments in mice

**Fig. 3:**
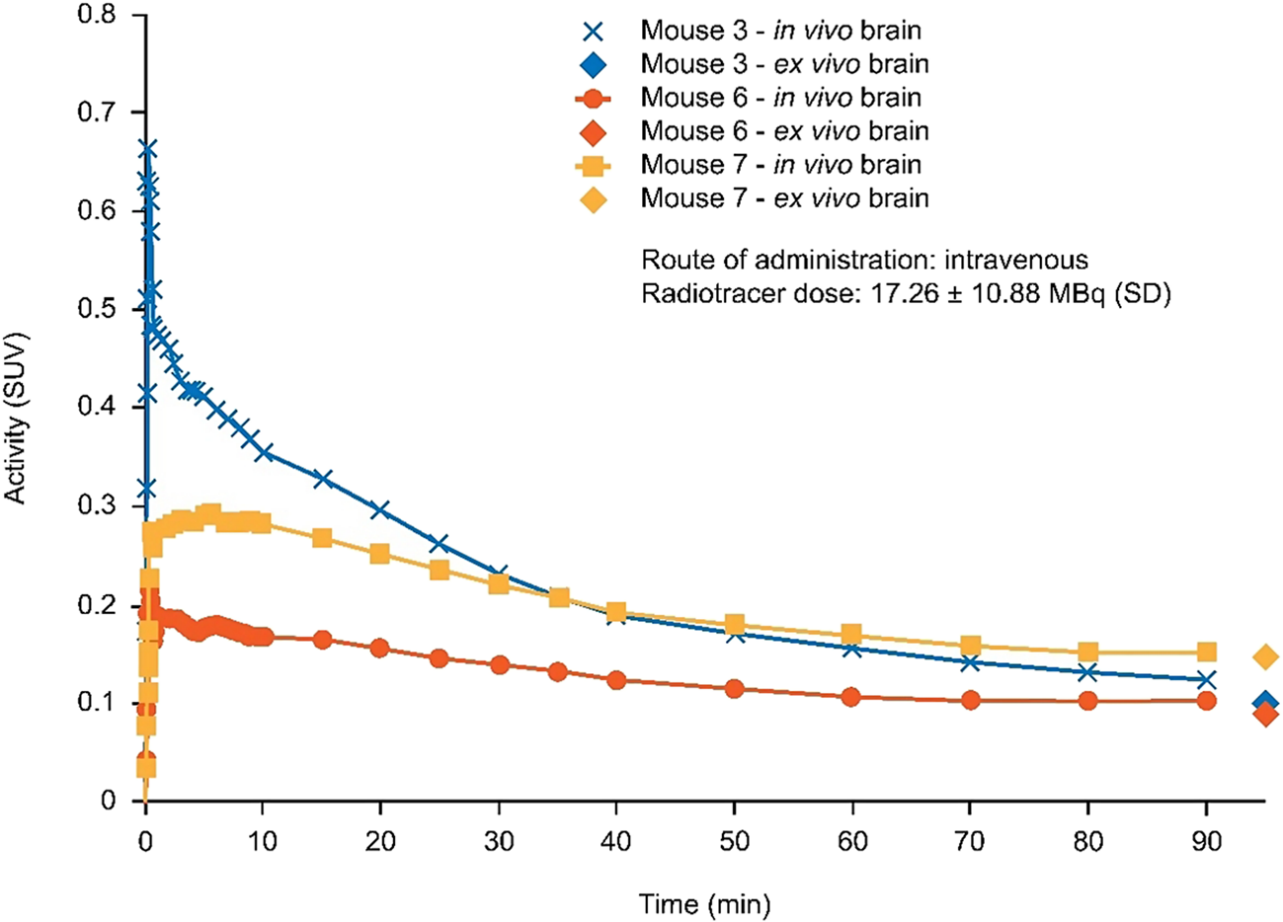
[^11^C]UCB2713 time activity curve in the brain (mouse) and *ex vivo* brain activity*. In vivo* experiments included PET/CT scan of the brain, performed on the three mice euthanized at 90 min post-administration of [^11^C]UCB2713; *ex vivo* experiments were performed on all 10 mice with samples of brain homogenate. *min* minute, *PET-CT* positron emission tomography-computed tomography, *SD* standard deviation, *SUV* standard uptake volume.

Time-activity curves for the whole brain (Fig. 3) showed low central nervous system (CNS) uptake with a standard uptake volume (SUV) of ~ 0.2 at 60 min. A good correlation between *in vivo* PET CNS SUVs and *ex vivo* SUVs was observed.

Radio-HPLC analysis of brain homogenate samples confirmed uptake of [^11^C]UCB2713 into the brain. No radio peaks for [^11^C-CO]UCB2713 were observed at the HPLC column eluent front from ~ 1.5–2 min, where polar metabolites are expected to elute, indicating there was no polar metabolite present in the mouse brain. Only a minor amount (~ 2%) of desmethyl-[^11^C-CO]UCB2713 was observed in mouse brain along with parent [^11^C-CO]UCB2713 (Supplemental Fig. 3a). In contrast, radio peaks for [^11^C-*N*-CH_3_]UCB2713 (Supplemental Fig. 3b) with a significant contribution of brain activity from polar metabolites were detected at ~ 1.5–2 min.

**Fig. 4:**
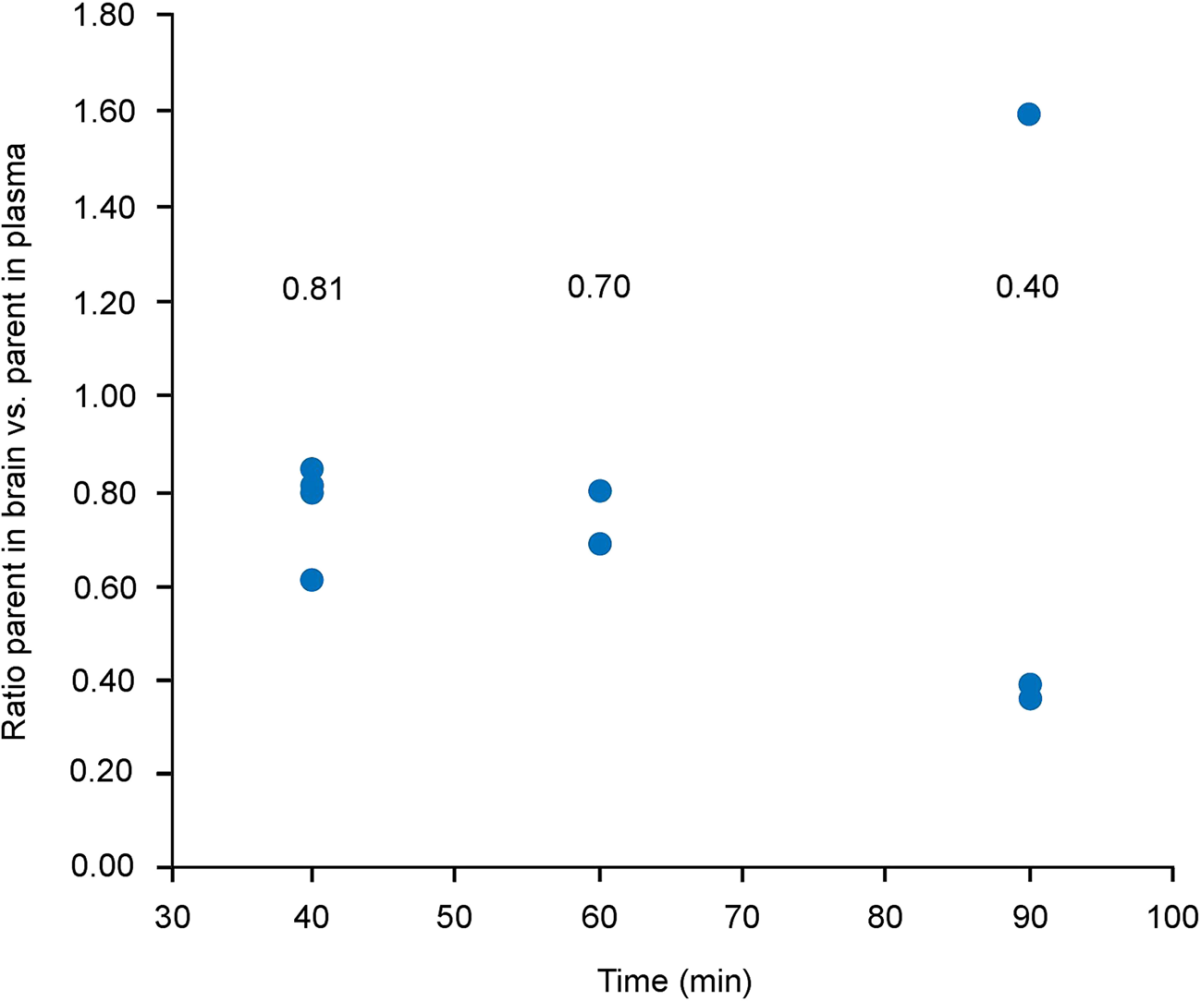
The ratio of parent ([^11^C]UCB2713) in brain:plasma over time. Median values are included above each group. *min* minute.

The ratio (SD) of the UCB2713 parent in the brain versus parent in plasma at time points 40 min (0.77 ± 0.11), 60 min (0.73 ± 0.06), and 90 min (0.79 ± 0.69), indicated successful uptake of [^11^C]UCB2713 into the brain (Fig. 4).

At 40 min post-administration of [^11^C]UCB2713, approximately 60% of the total brain activity and 30% of the total plasma activity corresponded to the parent, this decreased to approximately 30% and 20% at 90 min for the brain and plasma, respectively (Supplemental Fig. 4).

### Phase 1 PET Study in Healthy Male Participants (TM0017)

#### Study Demographics

A total of four participants were dosed with minzasolmin in the study and all participants completed the study. A summary of demographics is given in Table 2. No study participants took prior or concomitant medications. All participants received two radiotracer doses of [^11^C]minzasolmin (one prior to each of the two PET-CT scans) and a single oral dose of minzasolmin 360 mg (2 – 4 h prior to the second scan), in-house under medical supervision.

**Table 2:** TM0017—participant demographics

*N* = 4	Participant 1	Participant 2	Participant 3	Participant 4
Age (years)	30	47	26	28
Weight (kg)	86.4	72.1	73.1	73.7
Height (cm)	178	176	172	179
BMI (kg/m^2^)	27.3	23.3	24.7	23.0
Race	White	White	White	NC

*BMI* body-mass index, *NC* not collected

#### Brain Biodistribution of [11C]minzasolmin

**Fig. 5:**
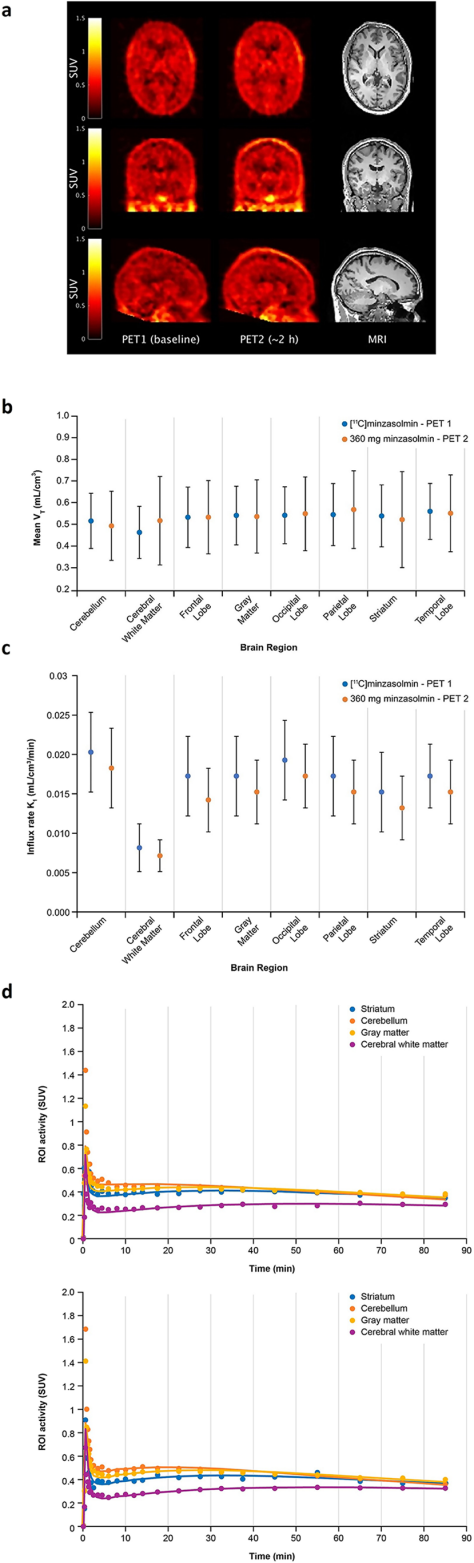
**a** TM0017—orthogonal cross-sections of co-registered PET and MR images from a representative participant. From left to right column images are PET Scan 1 (Baseline), PET Scan 2 (approximately 2 h post-dose), and structural (T1 weighted) MRI. The PET images are shown as SUV summed from 10 to 90 min. **b** Mean V_T_ of [^11^C]minzasolmin by selected brain region of participants in the TM0017 phase 1 study (error bars are one standard deviation from the mean). **c** Mean brain uptake rate for each brain region assessed from total plasma concentrations (least-squares mean, error bars are one standard deviation from the mean). Blue = PET Scan 1, orange = PET Scan 2. **d** TACs for PET Scan 1 (Baseline) and PET Scan 2 (approximately 2 h post-dose) for selected ROI (striatum, cerebellum, gray matter, and cerebral white matter). *h* hour*, K*_*1*_ mean brain influx rate, *min* minute, *MRI* magnetic resonance imaging, *PET* positron emission tomography, *ROI* region of interest, *SUV* standard uptake value, *T1* longitudinal relaxation time, *TAC* time activity curve, *V*_*T*_ the estimated whole brain total distribution volume at equilibrium.

A qualitative assessment of the PET images following radiotracer injections of [^11^C]minzasolmin showed the molecule was distributed throughout the brain (Fig. 5a). The highest concentrations of [^11^C]minzasolmin were seen in the cortical gray matter regions, striatum, and cerebellum. The mean V_T_ in all participants and across all ROI was 0.512 mL/cm^3^ (Fig. 5b); there was no difference in V_T_ observed before and after dosing with minzasolmin 360 mg. All ROI exhibited similar mean V_T_ values except for white matter, which was lower than most other ROI, with a mean of 0.491 mL/cm^3^. For Scan 1, there was a relatively large variation in V_T_ values across all ROI (Fig. 5b) which ranged from 0.341 to 0.657 mL/cm^3^. There was no dose effect on brain penetration of minzasolmin, as assessed by a mixed effects model, demonstrating there was no meaningful change in V_T_ across all ROI from PET Scan 1 to Scan 2 (the mean difference between Scan 1 and Scan 2 was -0.008 mL/cm^3^; estimated from the least squares mean of mixed model over all regions and participants).

The overall mean brain influx rate (K_1_) and brain efflux rate (k_2_) across each participant and all ROI was 0.015 and 0.029 mL/cm^3^/min, respectively. K_1_ was assessed from total plasma concentrations (i.e. no correction for plasma protein binding) and was between 0.007 and 0.022 mL/cm^3^/min (Fig. 5c); all ROI had similar mean K_1_ values, except for cerebral white matter (0.009 mL/cm^3^/min), which was considerably lower than other ROI. The mixed effects models showed small decreases in K_1_ and k_2_ (-15.9% and -9.3%, respectively) after oral dosing with minzasolmin 360 mg. Regional TACs were generated to include the striatum, cerebellum, gray matter, and cerebral white matter (Fig. 5d) and coefficient of variation (COV) of the estimates of K_1_ and V_T_ are shown in Table 3. Here, the average COV for V_T_ was 3.1%, while the average COV for K_1_ was 4.5%.

**Table 3:** Mean influx rate (K_1_), whole brain total distribution volume (V_T_) and coefficient of variation (COV) by selected brain region of participants in the TM0017 phase 1 study

Brain region	Scan	K_1_ (mL/cm^3^/min)	SD	COV (%)	V_T_ (mL/cm^3^)	SD	COV (%)
Cerebellum	1	0.02	0.005	5.8	0.522	0.127	3.2
	2	0.018	0.005		0.499	0.159	
Cerebral white matter	1	0.008	0.003	4.9	0.469	0.12	5.7
	2	0.007	0.002		0.523	0.204	
Frontal lobe	1	0.017	0.005	3.1	0.539	0.139	2.0
	2	0.014	0.004		0.539	0.169	
Gray matter	1	0.017	0.005	4.2	0.547	0.135	2.6
	2	0.015	0.004		0.542	0.169	
Occipital lobe	1	0.019	0.005	5.4	0.548	0.131	3.1
	2	0.017	0.004		0.555	0.17	
Parietal lobe	1	0.017	0.005	3.7	0.551	0.143	2.4
	2	0.015	0.004		0.574	0.179	
Striatum	1	0.015	0.005	3.7	0.545	0.143	2.6
	2	0.013	0.004		0.528	0.221	
Temporal lobe	1	0.017	0.004	4.7	0.566	0.129	3.1
	2	0.015	0.004		0.557	0.177	
Mean				4.5			3.1

*COV* coefficient of variation, *K*_*1*_, influx rate, *SD* standard deviation, *V*_*T*_ total distribution volume

In this study, arterial input function data were acquired and processed for all PET scans. Tracer metabolism was moderate, with ~ 50% of radioactivity in plasma at 90 min being attributable to intact parent radiotracer. The total parent fraction of [^11^C]minzasolmin was > 70% during the radiotracer brain uptake phase, up to 30 min (Supplemental Fig. 5).

#### Safety

In total, three TEAEs were reported by three out of four participants (75.0%), and none of the TEAEs were considered to be drug related. The three reported TEAEs (nasopharyngitis, back pain, and contact dermatitis) were mild in intensity. An overview of TEAEs by category is presented in Table 4. No clinically relevant findings in ECG parameters or vital sign measurements were observed. During the study, no deaths, serious AEs, or discontinuations were reported.

**Table 4:** TM0017—overview of TEAEs

	[^11^C]minzasolmin N = 4 n (%)	Minzasolmin 360 mg N = 4 n (%)
Any TEAEs	3 (75)	3 (75)
Serious TEAEs	0	0
Discontinuations due to TEAEs	0	0
Drug-related TEAEs	0	0
Severe TEAEs	0	0
TEAEs of special interest	0	0
Deaths	0	0

*TEAEs* treatment-emergent adverse events

## Discussion

The preclinical evaluation and optimization focused on the *S*-enantiomer of minzasolmin (UCB2713). Two potential radiolabeling positions were evaluated, [^11^C-CO]UCB2713 and [^11^C-*N*-CH_3_]UCB2713, to optimize [^11^C]minzasolmin for human studies. Both radiolabeling positions of UCB2713 were feasible, though the radiochemical yield of [^11^C-CO]UCB2713 was low, relative to [^11^C-*N*-CH_3_]UCB2713. *In-vivo* metabolism studies of [^11^C-CO]UCB2713 in mice indicated > 95% of the parent fraction was in brain tissue, whereas the [^11^C-*N*-CH_3_]UCB2713 fraction in the brain was markedly lower. The analysis of the [^11^C-*N*-CH_3_]UCB2713 time course indicated metabolism was slow. The polar metabolites that were observed are thought to be polar radiolabeled species and may arise from *N*-[^11^C]methyl bond cleavage, leading to the formation of [^11^C]HCHO, [^11^C]HCOOH, [^11^C]CO_2_ [16]. In the TM0017 study, tracer metabolism was moderate and much slower than in mouse, with ~ 50% of radioactivity in plasma at 90 min being attributable to intact parent radiotracer; total parent fraction of [^11^C]minzasolmin was > 70% during the brain uptake phase, up to 30 min. Given that the rate of tracer metabolism is much lower in humans compared with mice, it would not be useful in this context to include the mouse tracer metabolism data, and furthermore, the metabolites were found to be below the lower limit of quantitation in the UP0030 study [10].The UP0030 study also showed that the concentrations of both the *N*-desmethyl and the N-oxide metabolites in CSF of study participants were very low 2 h post dose (~ 20 times lower than the parent minzasolmin concentration) [10]. Based on the mouse data in the preclinical study, we selected [^11^C-*N*-CH_3_]-labeled rather than [^11^C-CO]-labeled minzasolmin for the TM0017 human study.

In the phase 1 PET study (TM0017) in healthy males, minzasolmin distributed throughout the brain and was observed in all gray and white matter regions. The overall mean V_T_ values were approximately 0.5 mL/cm^3^, indicating that at steady-state total brain tissue concentrations would be around half (total brain to total plasma concentration, partition coefficient; Kp of ~ 0.5) of the corresponding total plasma concentration, which represents good CNS entry. This finding is much higher than would be expected with the regional intravascular blood volume component (approximately 5%) of the PET signal, indicating that [^11^C]minzasolmin crosses the blood–brain barrier. Based on the known total plasma concentration, these results in addition to results from a previous study with minzasolmin (UP0077) suggest that the maximum projected human total brain concentration of up to 1273 ng/mL (from a minzasolmin 360 mg/day dose) can be achieved [10]; this total brain concentration is higher than the concentration which showed efficacy in a pre-clinical mouse model of PD [8].

Overall, there was no difference in V_T_ across all ROI following oral administration of minzasolmin 360 mg versus following IV radiotracer dose administration of [^11^C]minzasolmin and it can therefore be expected that V_T_ will be constant within the radiotracer (≤ 10 μg) to 360 mg range. This also confirms the expectation for a compound that is not affected by efflux pump activity [10]. The K_1_ values calculated from total plasma concentrations were between 0.007 and 0.022 mL/cm^3^/min, with a plasma-free fraction of ~ 2.5%, which is consistent with high extraction and penetration of the blood–brain barrier. Since the mechanism of action of minzasolmin is located at the cell membrane [9], and Kp uu for minzasolmin has already been determined [10], we did not sample CSF to determine the Kp uu in this study. We hypothesized that the ratio of total minzasolmin in the cortex to free minzasolmin in plasma could be much greater than the ratio of CSF to free plasma minzasolmin, and could be studied non-invasively with PET.

Whilst pharmacokinetic (PK) data are limited in this small cohort study, findings were consistent with other larger phase 1/1b studies investigating minzasolmin. PK data for TM0017 and UP0030 following administration of single dose minzasolmin up to 450 mg are shown in Table 5. The apparent terminal elimination half-life measured in this phase 1 PET study was 11.43 h (GeoMean), which was also consistent with the previous phase 1/1b studies (GeoMean range: 9.3–13.1 h) [10].

**Table 5:** Pharmacokinetic parameters for minzasolmin in the TM0017 and UP0030 (NCT04875962) studies [10]

Parameter statistic	TM0017 minzasolmin 360 mg	Parameter statistic	UP0030 minzasolmin 90–450 mg (single ascending dose study)
C_max_ (ng/mL)		C_max_ (ng/mL)	
GeoMean	1580	GeoMean	374–1806
t_max_ (h)		t_max_ (h)	
Median	2	Median	1.5–2
AUC (h*ng/mL)		AUC (h*ng/mL)	
GeoMean	13230	GeoMean	3225–17040

*AUC* area under the plasma concentration–time curve from time zero to infinity, *C*_*max*_ maximum plasma concentration, *GeoMean* geometric mean, *h* hours, *t*_*max*_ time to maximum plasma concentration

There were no new safety signals reported in this study [10], and there were no clinically relevant findings in laboratory parameters, vital signs, or ECG recordings. A limitation of the study is the small sample size of four participants, however, safety data reported are consistent with other studies which investigated the safety of minzasolmin at the same dosage (360 mg) in larger populations in healthy participants and people living with PD [10].

ASYN is a genetically validated target in PD [17] and therapies targeting ASYN pathology have the potential to be disease-modifying, slowing/halting the progression of PD [8]. However, recent studies of ASYN monoclonal antibodies have failed to meet their primary endpoints [18, 19]; potential reasons for failure include: study design, lack of target engagement, and insufficient dose levels reached in target tissue [20]. Here we show that minzasolmin, an oral small molecule inhibitor of ASYN misfolding, is distributed in both gray and white matter regions of the brain and is able to reach the brain at a concentration similar to the concentration that demonstrated efficacy in a mouse model of PD.

## Conclusions

**Fig. 6:**
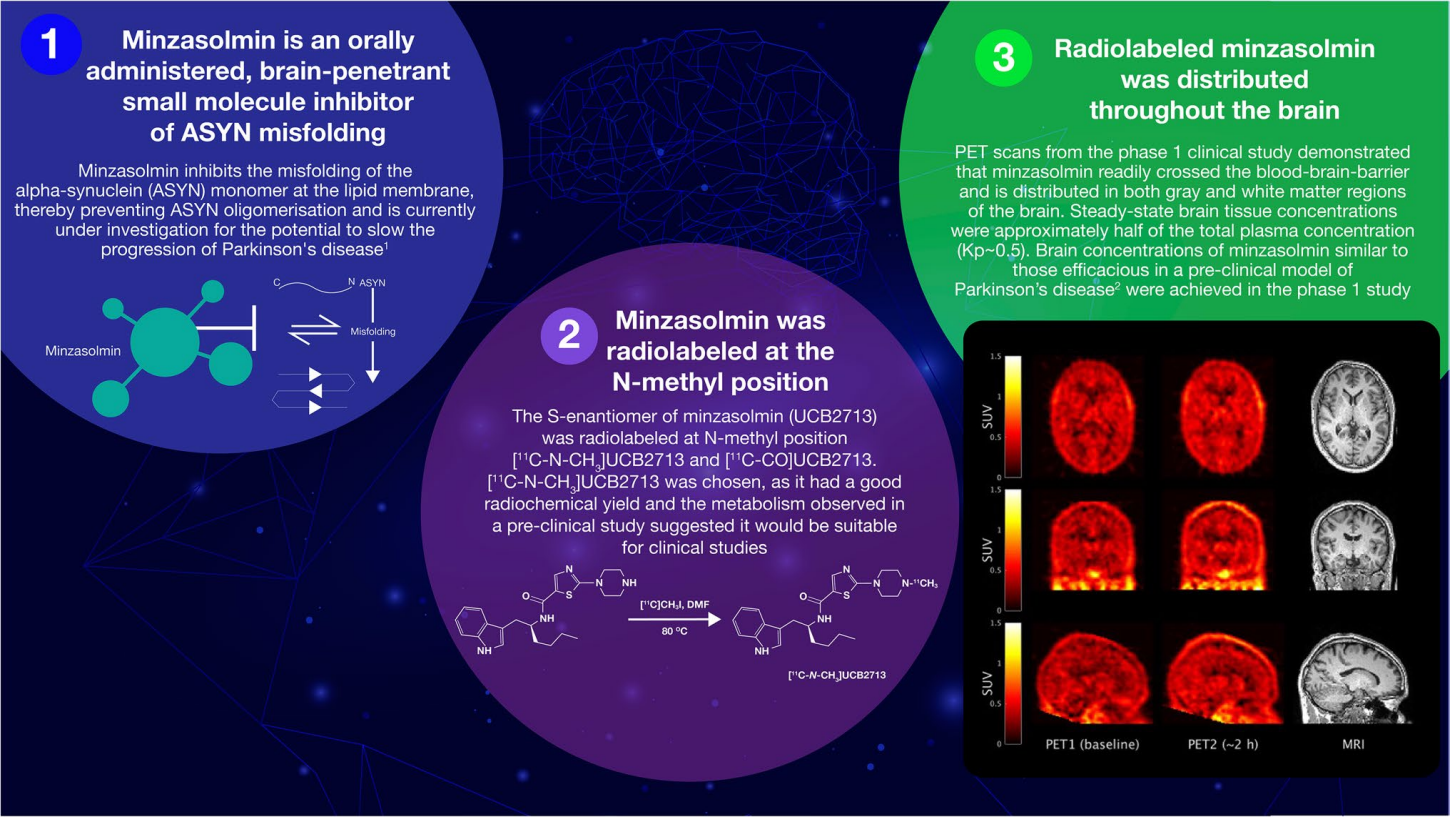
Summary of the preclinical and clinical studies *ASYN* alpha-synuclein, *h* hour, *K*_*p*_ partition coefficient, *MRI* magnetic resonance imaging, *PET* position emission tomography, *SUV* standard uptake value.

The preclinical study allowed for selection of a radiolabeled ligand that would perform well in the phase 1 study. In the phase 1 study, minzasolmin was orally administered in healthy participants; minzasolmin demonstrated clear and fast brain penetration and readily crossed the blood–brain barrier, where steady-state brain tissue total concentrations were approximately half of the total plasma concentrations. Total brain concentrations similar to those that demonstrated efficacy in a mouse model of PD were achieved in the phase 1 study, after a single oral dose of minzasolmin 360 mg (Fig. 6). All safety and PK findings following oral administration of minzasolmin were consistent with previous phase 1/1b studies. Together, the findings of these studies support the further clinical development of minzasolmin as a potential disease-modifying therapy for PD.

### Supplementary Information

Below is the link to the electronic supplementary material.

Supplementary file1 (PDF 703 KB)

## Data Availability

Data from non-clinical studies is outside of UCB’s data sharing policy and is unavailable for sharing*.* Due to the small sample size in this trial, individual patient-level data cannot be adequately anonymized as there is a reasonable likelihood that individual participants could be re-identified. For this reason, data from this trial cannot be shared.
