# Supplementary material for: Evaluation and Application of a PET Tracer in Preclinical and Phase 1 Studies to Determine the Brain Biodistribution of Minzasolmin (UCB0599)
Source: Mol Imaging Biol. 2023 Dec 18;26(2):310–21. doi: 10.1007/s11307-023-01878-7 (PMC10973027; doi:10.1007/s11307-023-01878-7)
Supplement: Supplementary file 1 — Supplementary file1 (PDF 703 KB) [file 11307_2023_1878_MOESM1_ESM.pdf]

Electronic Supplementary Material

Evaluation and Application of a PET Tracer in Preclinical and Phase 1 Studies to Determine the Brain Biodistribution of Minzasolmin (UCB0599)

Running Title: PET Tracer to Determine Brain Biodistribution of Minzasolmin

Joël Mercier[1], Massimo Bani[1], Anny-Odile Colson[1], Massimiliano Germani,[1] Marianna Lalla[1]*, Christophe Plisson[2], Mickael Huiban[2], Graham Searle[2], François-Xavier Mathy[1], Richard Nicholl[3], Christian Otoul[1], Johan Willem Smit[1†], Vanja van Asch[4], Michel Wagneur[1], and Ralph Paul Maguire[1]

[1]*UCB Pharma, Braine l'Alleud, Belgium*

[2]*Invicro, London, UK*

[3]*UCB Pharma, Slough, UK*

[4]*Auximines Clinical Solutions, Zemst, Belgium*

**Current affiliation: OxSonics, Oxford, UK*

[†]*Current affiliation: Curare Consulting, Hamburg, Germany*

Corresponding Author

Dr Ralph Paul Maguire, ORCID ID 0000-0003-3937-6622

Email: paul.maguire@ucb.com

Telephone: +32 (0)2 386 4968

Methods

Synthesis of precursor Compounds 1 – 3

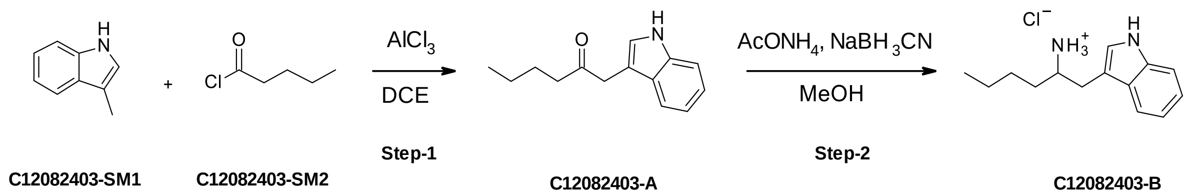

<u>1-(1H-indol-3-yl)hexan-2-amine (</u><u>C12082403-B Lot BF14001</u>**<u>)</u>**

To a solution of 3-methyl-1H-indole (14.5 kg, 1 Eq.) in dichloroethane (241 kg) at 0° C, $AlCl_3$ (46.1 kg, 3 Eq.) was added. The reaction mixture (RM) was heated to room temperature (RT) for 5 hours and then cooled again to 0° C. Pentanoyl chloride (13.4 kg, 0.9 Eq.) was added dropwise under nitrogen and the RM was stirred at 0° C for 30 min and at RT for 13 hours. $AlCl_3$ (14.8 kg, 1 Eq.) was added again at 0° C. The RM was heated to RT for 5 hours then cooled again to 0° C and pentanoyl chloride (6.8 kg, 0.45 Eq.) was added dropwise under nitrogen and the RM was stirred at 0° C for 30 min and at RT for 20 hours. The reaction was stopped by pouring the RM into water (220 kg) maintained at 0–3° C and then extracted with dichloromethane (DCM) (4 x 197 kg). The combined organic layers were washed twice with an aqueous solution of $NaHCO_3$ (7%, 150 kg), once with an aqueous solution of NaCl (25%, 150 kg) and then concentrated under vacuum. Methanol (99 kg) was added twice to the crude material which was further concentrated under vacuum. The resulting crude mixture was then dissolved in MeOH (60 kg) to afford 106 kg of a solution of C12082403-A that was used as such in the next step.

C12082403-A in MeOH (12.7 kg) was added to ammonium acetate (47.5 kg, 3.5 Eq.) in anhydrous MeOH (80 kg). Sodium cyanoborohydride (5.7 kg,0.45 Eq.) was then added. The

RM was heated to 60~75 °C for 24 hours and then cooled to 45 °C and concentrated under vacuum.

DCM (133 kg) was added to the residue and then concentrated under vacuum. This process is repeated 3 times and then DCM (198 kg) is added again followed by water (62 kg) and an aqueous solution of Sodium hydroxide (30%, 33 kg). The heterogeneous mixture is stirred for 50 min and then allowed to stand for an additional 50 min.

The organic layer was separated, and the aqueous layer extracted one more time with DCM (120 kg). The combined organic phases are then washed with an aqueous NaCl solution (25%, 66 kg) and stirred with HCl (2M in water, 10 kg) for 50 min. The organic layer is separated and the washing steps with water (66 kg) and NaCl solution (66 kg) are repeated. The organic phase is concentrated under vacuum to afford crude C12082403-B (15.8 kg). The crude material is then purified by adding isopropyl acetate (55 kg) and MeOH (12.6 kg) and refluxing at 65~75 °C for 24 hours. The solid material is then filtered and rinsed with methyl tert-butyl ether (MTBE) (48 kg) to afford C12082403-B lot BF14001 (9.06 kg) as a hydrochloride salt. HPLC (basic condition): 98% purity.

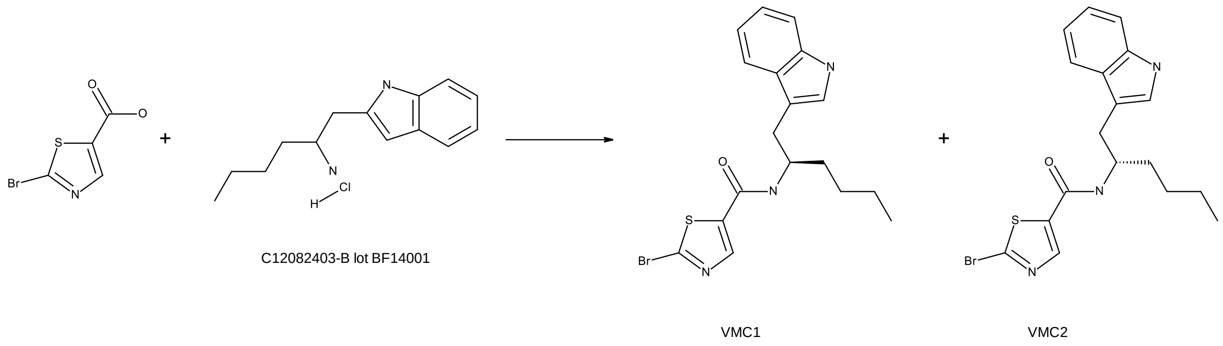

2-bromo-N-[(1R)-1-(1H-indol-3-ylmethyl)pentyl]thiazole-5-carboxamide (VMC1) and 2-bromo-N-[(1S)-1-(1H-indol-3-ylmethyl)pentyl]thiazole-5-carboxamide (VMC2)

To a solution of 2-bromothiazole-5-carboxylic acid (Interchim, 5.2 g, 25 mmol) and 1-(1H-indol-3-yl)hexan-2-amine hydrochloride (C12082403-B lot BF14001, 5.3 g, 21 mmol) in

tetrahydrofuran (100 mL, 1233 mmol) and water (10 mL) was added 1-(3-dimethylaminopropyl)-3-ethylcarbodiimide hydrochloride (5.3 g, 27 mmol) and 1-hydroxybenzotriazole hydrate (0.8 g, 5 mmol). The reaction mixture was cooled to 0 °C and 4-methylmorpholine (10 mL, 90 mmol) was added. The reaction mixture was stirred at room temperature for 18 hours. The progress of the reaction was monitored by liquid chromatography mass spectrometry (LCMS). After completion of reaction, the reaction mixture was diluted with AcOEt (200 mL), washed with water (2x200 mL), with a saturated solution of $NaHCO_3$ (2x200 mL), with a solution of HCl 1M (2x200 mL) and with water (1x200 mL). The organic layer was dried over $MgSO_4$, filtered and concentrated under reduced pressure.

The chiral separation was performed by supercritical fluid (SFC) (column Lux Cellulose-2, elution CO_2+20%EtOH) to afford 2-bromo-N-[(1R)-1-(1H-indol-3-ylmethyl)pentyl]thiazole-5-carboxamide VMC1 as a yellow solid (2.67 g, yield: 38.4%) and 2-bromo-N-[(1s)-1-(1H-indol-3-ylmethyl)pentyl]thiazole-5-carboxamide VMC2 as a yellow solid (3.03 g, yield: 43.6%.

LCMS (basic condition): $[M+H]^+$ m/z 406/408, RT 2.68 min.

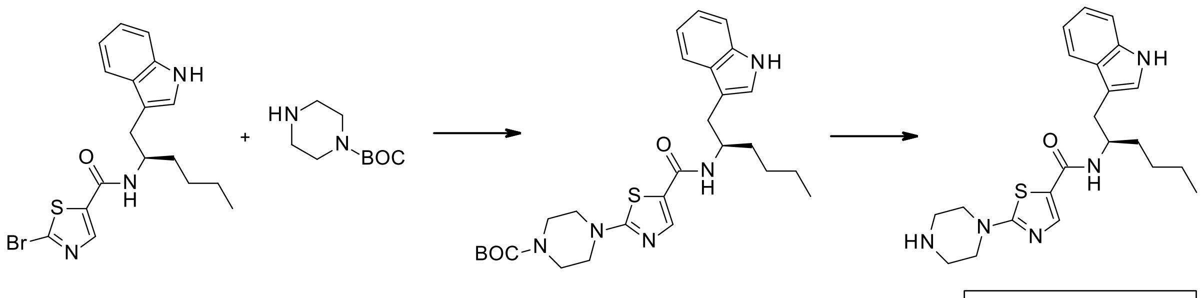

VMC1

VMC3

Compound 1

tert-butyl 4-[5-[[(1R)-1-(1H-indol-3-ylmethyl)pentyl]carbamoyl]thiazol-2-yl]piperazine -1-carboxylate (VMC3)

To a solution of 2-bromo-N-[(1R)-1-(1H-indol-3-ylmethyl)pentyl]thiazole-5-carboxamide (VMC1, 2.67 g, 6.611 mmol) in acetonitrile (2 mL, 38.2948 mmol) was added potassium carbonate (2.31 g, 16.5 mmol) and 1-boc-piperazine (B, 2.51 g, 13.2 mmol). The reaction mixture was stirred at 80°C for 18 hours. The progress of the reaction was monitored by LCMS. After completion of reaction, the reaction mixture was evaporated under reduce pressure. The residue was diluted with AcOEt (200 mL), washed with a saturated solution of NaCl (2x100 mL). The organic layer was dried over $MgSO_4$, filtered and concentrated under reduced pressure to afford tert-butyl 4-[5-[[(1R)-1-(1H-indol-3-ylmethyl)pentyl]carbamoyl]thiazol-2-yl]piperazine-1-carboxylate as a yellow oil (2.99 g, yield: 88%).

LCMS (basic condition): $[M+H]^+$ m/z 512, RT 2.88 min.

N-[(1R)-1-(1H-indol-3-ylmethyl)pentyl]-2-piperazin-1-yl-thiazole-5-carboxamide (Compound 1)

Tert-butyl 4-[5-[[(1R)-1-(1H-indol-3-ylmethyl)pentyl]carbamoyl]thiazol-2-yl]piperazine-1-carboxylate (VMC3, 2.99 g, 5.84 mmol) was dissolved in hydrochloric acid 4M in dioxane (100 mL, 400 mmol). The reaction mixture was stirred at room temperature during the weekend. The reaction was monitored by LCMS. After completion of reaction, the solid was filtered and dried under reduce pressure for 18 hours. The solid was dissolved in AcOEt (200 mL), basified with a saturated solution of $NaHCO_3$ and extracted with a saturated solution of $NaHCO_3$ (2x100 mL). The organic layer was dried over $MgSO_4$, filtered and concentrated under reduced pressure, the solid was purified by reverse phase chromatography in basic condition to afford N-[(1R)-1-(1H-indol-3-ylmethyl)pentyl]-2-piperazin-1-yl-

thiazole-5-carboxamide as a white solid (323 mg, yield: 13%)

LCMS (basic condition): $[M+H]^+$ m/z 412, RT 5.65 min.

LCMS (acidic condition): $[M+H]^+$ m/z 412, RT 4.25 min.

LC chiral (LuxCell2 EtOH 50% - heptane 50% - DEA 0.1% 30°C : peak@3.76 min, ee 99%

^{1}H NMR (400 MHz, DMSO) δ 10.75 (d, *J* = 2.3 Hz, 1H), 7.92 (d, *J* = 8.5 Hz, 1H), 7.80 (s, 1H), 7.58 (d, *J* = 7.8 Hz, 1H), 7.31 (d, *J* = 8.0 Hz, 1H), 7.09 (d, *J* = 2.3 Hz, 1H), 7.04 (ddd, *J* = 8.1, 7.0, 1.2 Hz, 1H), 6.96 (ddd, *J* = 7.9, 7.0, 1.0 Hz, 1H), 4.19 – 4.04 (m, 1H), 3.39 – 3.33 (m, 4H), 2.94 – 2.74 (m, 6H), 1.60 – 1.42 (m, 1H), 1.30 – 1.12 (m, 2H), 0.81 (t, *J* = 6.8 Hz, 3H).

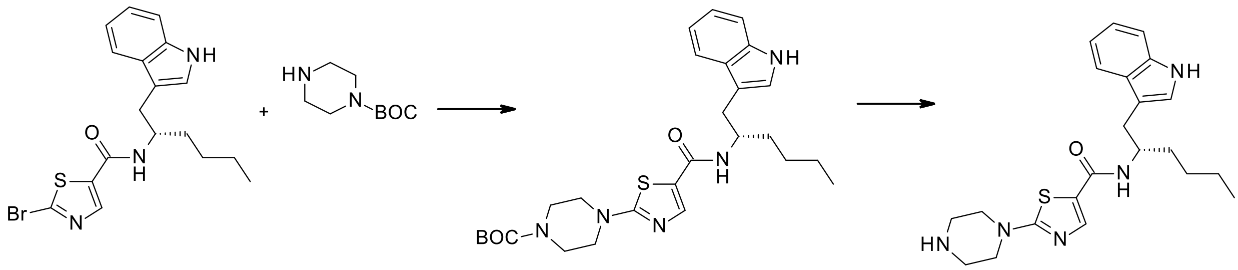

VMC2 VMC5 VMC6

<u>Tert-butyl 4-[5-[[(1S)-1-(1H-indol-3-ylmethyl)pentyl]carbamoyl]thiazol-2-yl]piperazine-1-carboxylate (VMC5)</u>

To a solution of 2-bromo-N-[(1S)-1-(1H-indol-3-ylmethyl)pentyl]thiazole-5-carboxamide (VMC2, 2.374 g, 5.843 mmol) in acetonitrile (2 mL, 38.2948 mmol) was added potassium carbonate (2.04 g, 14.6 mmol) and 1-boc-piperazine (B, 2.22 g, 11.7 mmol). The reaction mixture was stirred at 80 °C for 18 hours. The progress of the reaction was monitored by LCMS. After completion of reaction, the reaction mixture was evaporated under reduce pressure. The residue was diluted with AcOEt (200 mL), washed with a saturated solution of NaCl (2x100 mL). The organic layer was dried over $MgSO_4$, filtered and concentrated under reduced pressure to afford tert-butyl 4-[5-[[(1S)-1-(1H-indol-3-

ylmethyl)pentyl]carbamoyl]thiazol-2-yl]piperazine-1-carboxylate as a yellow oil (2.63 g, yield: 88%).

LCMS (basic condition): $[M+H]^+$ m/z 512, RT 2.91 min.

N-[(1S)-1-(1H-indol-3-ylmethyl)pentyl]-2-piperazin-1-yl-thiazole-5-carboxamide (VMC6)

Tert-butyl 4-[5-[[(1S)-1-(1H-indol-3-ylmethyl)pentyl]carbamoyl]thiazol-2-yl]piperazine-1-carboxylate (VMC5, 2.63 g, 5.14 mmol) was dissolved in hydrochloric acid 4M in dioxane (100 mL, 400 mmol). The reaction mixture was stirred at room temperature during the weekend. The reaction was monitored by LCMS. After completion of reaction, the solid was filtered and dried under reduce pressure for 18 hours. The solid was dissolved in AcOEt (200 mL), basified with a saturated solution of $NaHCO_3$ and extracted with a saturated solution of $NaHCO_3$ (2x100 mL). The organic layer was dried over $MgSO_4$, filtered and concentrated under reduced pressure, the solid was purified by reverse phase chromatography in basic condition to afford N-[(1S)-1-(1H-indol-3-ylmethyl)pentyl]-2-piperazin-1-yl-thiazole-5-carboxamide as a white solid (340 mg, yield: 14%).

LCMS (basic condition): $[M+H]^+$ m/z 412, RT 5.63 min.

LCMS (acidic condition): $[M+H]^+$ m/z 412, RT 4.28 min.

LC chiral (LuxCell2 EtOH 50% - heptane 50% - DEA 0.1% 30°C): peak @2.56 min, ee 96%

^{1}H NMR (400 MHz, DMSO) δ 10.75 (s, 1H), 7.91 (d, *J* = 8.5 Hz, 1H), 7.80 (s, 1H), 7.57 (d, *J* = 7.8 Hz, 1H), 7.31 (d, *J* = 8.0 Hz, 1H), 7.09 (d, *J* = 2.3 Hz, 1H), 7.04 (ddd, *J* = 8.1, 7.0, 1.2 Hz, 1H), 6.95 (td, *J* = 7.4, 1.1 Hz, 1H), 4.11 (d, *J* = 5.0 Hz, 1H), 3.37 – 3.33 (m, 5H),

2.95 – 2.79 (m, 2H), 2.80 – 2.73 (m, 4H), 1.60 – 1.42 (m, 1H), 1.29 – 1.13 (m, 2H), 0.87 – 0.74 (m, 3H).

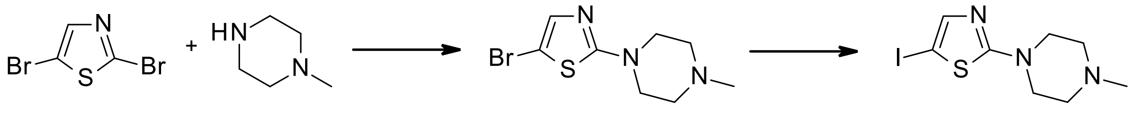

VMC7 **Compound 2**

5-bromo-2-(4-methylpiperazin-1-yl)thiazole (VMC7)

To a solution of 2,5-dibromothiazole (ALFA batch 10151493, 5 g, 19.9654 mmol) in acetonitrile (20 mL, 382.9476 mmol) was added 1-methylpiperazine (ACROS batch A011376901, 4 mL, 40 mmol) and potassium carbonate (7 g, 50.1411 mmol). The reaction mixture was stirred at 80 °C during 4h. The progress of the reaction was monitored by LCMS. After completion of reaction, the reaction mixture was diluted with AcOEt (200 mL), washed with water (2x100 mL). The organic layer was dried over $MgSO_4$, filtered and concentrated under reduced pressure. The residue was purified by reverse phase chromatography in basic condition to afford 5-bromo-2-(4-methylpiperazin-1-yl)thiazole as an off-white solid (2.66 g, yield: 50%).

LCMS (basic condition): $[M+H]^+$ m/z 262/264, RT 2.09 min.

^{1}H NMR (400 MHz, DMSO) δ 7.49 (s, 1H), 3.68 – 3.61 (m, 5H), 2.72 – 2.65 (m, 4H).

5-iodo-2-(4-methylpiperazin-1-yl)thiazole (Compound 2)

To a solution of 5-bromo-2-(4-methylpiperazin-1-yl)thiazole (VMC7, 200 mg, 0.76286 mmol) in 1,4-dioxane (5 mL, 58.6142 mmol) was added sodium iodide (686 mg, 4.5766 mmol), cuprous iodide (28 mg, 0.147 mmol) and N,N'-dimethylethylenediamine (17 µL, 0.1564 mmol). The reaction mixture was stirred at 80°C during the weekend. The progress of

the reaction was monitored by LCMS. After completion of reaction, the reaction mixture was diluted with AcOEt (50 mL), washed with a solution of $Na_2S_2O_3$ (2x30 mL). The organic layer was dried over $MgSO_4$, filtered and concentrated under reduced pressure to afford 5-iodo-2-(4-methylpiperazin-1-yl)thiazole (187 mg, yield: 79%).

LCMS (basic condition): $[M+H]^+$ m/z 310, RT 2.14 min.

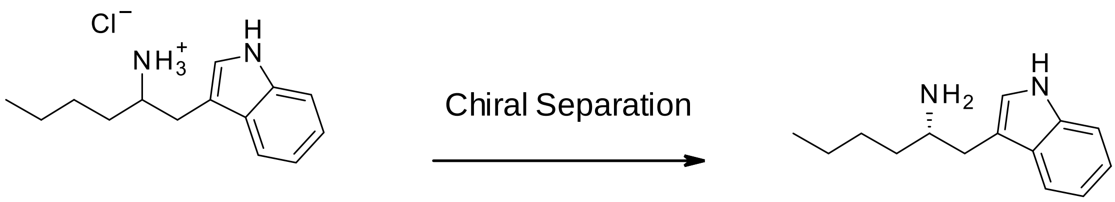

C12082403-B **Compound 3**

(2S)-1-(1H-indol-3-yl)hexan-2-amine (Compound 3)

A chiral chromatography of C12082403-B Lot BF14001 was performed on Chiralcel OJ-H using MeOH 100% containing 0.1% of diethylamine as eluent to afford (2S)-1-(1H-indol-3-yl)hexan-2-amine as the first eluted compounds.

LC chiral (Chiralcel OJ-H MeOH 100% - DEA 0.1% 30 °C : peak @4.21 min, ee 99%

Preparation of [^{11}C-N-CH$_3$]UCB2713

[^{11}C]carbon dioxide was produced by the $^{14}N(p,\alpha)^{11}C$ nuclear reaction using a nitrogen gas target (containing 1% oxygen) pressurized to 300–350 psi and bombarded with 11 MeV protons using the Siemens RDS-111 Eclipse cyclotron (Siemens, Munich, Germany). Subsequently, [^{11}C]carbon dioxide was converted into [^{11}C]methyl iodide by catalytic reduction (H_2/Ni) which gave the [^{11}C]methane intermediate followed by gas phase

iodination with iodine. [^{11}C]methyl iodide was delivered to a solution of precursor (Compound 1) (1.00 mg, 2.43 µmol) in dimethylformamide (400 µL) at 15 °C using a helium stream. After delivery of [^{11}C]iodomethane, the sealed vessel was heated to 80 °C for 5 min. The reaction mixture was then cooled down to 20 °C for 2 min, diluted with 1 mL of high-performance liquid chromatography (HPLC) eluent and loaded onto the HPLC injection loop for purification. The HPLC conditions employed for the purification of [^{11}C-*N*-CH_3]UCB2713 were as follows:

- Column Agilent XDB-C18, 5 µm, 250 x 9.4 mm
- Flow rate: 9.5 $mL.min^{-1}$
- Ultraviolet (UV) wavelength: 254 nm
- Mobile phase
  - Eluent A: ammonium formate buffer 100 mM pH 8
  - Eluent B: acetonitrile/H_2O 95/5
- Isocratic conditions: eluent A/eluent B: 52/48

The semi-preparative HPLC fraction corresponding to [^{11}C-*N*-CH_3]UCB2713 eluting at 7.9 min was collected, diluted with 20 mL of water, and passed through a Strata C18 cartridge (Strata-X 33µm Polymeric reversed phase [Phenomenex, Torrance CA, USA]). The Strata cartridge was washed with 5 mL of water. The purified [^{11}C-*N*-CH_3]UCB2713 was eluted from the column with 0.5 mL of absolute ethanol and recovered in a sterile vial. Thereafter, 4.5 mL of 0.9% saline solution was passed through the Strata cartridge and collected in the sterile vial. Finally, the dose was mixed by nitrogen bubbling for 5 seconds.

Quality control and determination of the molar activity of [^{11}C-*N*-CH_3]UCB2713 were performed by analytical HPLC (Supplemental Fig. 1 and 2),

using an Eclipse XDB-C18 column (4.6 x 150 mm, 5 µm). Isocratic elution was performed using a mixture of ammonium formate buffer 50 mM pH 8 (solvent A) and CH_3CN (solvent B) 55/45 at a flow rate of 1.5 mL/min^{-1}.

Preparation of [^{11}C-CO]UCB2713

Preparation of the [^{11}C]carbonylation Reagents (Typical Quantities)

In a 5 mL V-bottomed glass vial were added the iodo precursor (Compound 2: 5-iodo-2-[4-methylpiperazin-1-yl]thiazole, SN145369/004) (1.01 mg, 3.25 µmol), the amino precursor ([2S]-1-[1*H*-indol-3-yl]hexan-2-amine, B2270/084) (3.52 mg, 16.3 µmol), the Pd species Pd(π-cinnamyl)Cl_2 (0.83 mg, 1.59 µmol) and the ligand Xantphos (2.47 mg, 4.28 µmol). The reagents were flushed with argon for 10 min followed by addition of anhydrous *N*,*N*-dimethylformamide (400 µL) and Et_3N (10 µL) and finally flushed with argon for 5 min.

Preparation of [^{11}C]carbon monoxide

[^{11}C]carbon dioxide was produced using a Siemens Eclipse HP cyclotron by 11 MeV proton bombardment of a target containing nitrogen and 1% oxygen. [^{11}C]carbon monoxide was produced using an Eckert and Ziegler reduction module. [^{11}C]carbon dioxide was delivered from the cyclotron and trapped at room temperature in a stainless-steel loop containing molecular sieves. The [^{11}C]carbon dioxide was then released by passing a helium flow through the loop while heating to 400 °C. The resultant [^{11}C]carbon dioxide/helium gas stream was passed through a glass tube packed with molybdenum powder at 850 °C, converting the [^{11}C]carbon dioxide to [^{11}C]carbon monoxide with any unreduced [^{11}C]carbon dioxide being trapped using Ascarite®. The resultant [^{11}C]carbon monoxide/helium gas stream was delivered to the reaction vial at a flow rate of

10 mL/min. The time taken from end of cyclotron bombardment to complete delivery of $[^{11}C]$carbon monoxide to the reaction vial was 5 to 6 min.

$[^{11}C]$carbonylation

$[^{11}C]$carbon monoxide/helium gas stream was delivered to the 5 mL V-bottomed glass vial containing the carbonylation reagents at 120 °C (the reactor was heated to 120 °C in parallel with heating of the $[^{11}C]$carbon dioxide trap to 400 °C). The unreacted $[^{11}C]$carbon monoxide was captured on a trap filled with carbosphere, preceded of a stainless-steel loop, both cooled down in a Dewar full of liquid Nitrogen. The waste gas was collected in a bag placed after the carbosphere trap. The radioactivity was monitored in the reaction vial and on the trap using the pin diodes, and in the waste gas using a dose calibrator (Capintec, Florham Park, NJ, USA). When the radioactivity in the vial and the trap reached a maximum, the system was closed. The trap filled with carbosphere was lifted up whereas the empty loop stayed in liquid nitrogen in order to capture any residual dimethylformamide. Thereafter, the trap was heated for a few seconds with a heat gun. Then, the recirculation pump was started to initiate the recirculation of $[^{11}C]$carbon monoxide through the reactor (maintained at 120 °C). The reaction mixture was then cooled down to 20 °C for 1 min. Thereafter, the reaction mixture was diluted with 1 mL of HPLC eluent and loaded onto the HPLC injection loop for purification. The purification and quality control of $[^{11}C\text{-CO}]$UCB2713 were performed as previously described for $[^{11}C\text{-}N\text{-}CH_3]$UCB2713.

[^{11}C]UCB2713 in vivo/ex vivo Evaluation

Administration of Radioligand and Collecting Samples

Each mouse received an intravenous (IV) bolus injection of [^{11}C]UCB2713 via the jugular vein. The mean (standard deviation [SD]) mass of each radiotracer dose was 1.54 (± 1.03) µg; with a radioactive dose of 17.26 (± 10.88) MBq. At pre-defined time points (40, 60, and 90 min) post-administration of [^{11}C]UCB2713, each mouse was euthanized by exsanguination, followed by cervical dislocation, with blood samples collected in heparin tubes and the brain dissected.

Brain Sample Processing and Preparation of Sample for Analysis

A sample of brain homogenate (1:1, brain:water) was assessed for radioactivity using a γ-counter. Brain homogenate was extracted with a ratio of 1:2 (homogenate:acetonitrile); a ratio of 1:1 was used for the first two animals. The mixture was vortexed for 3 min followed by centrifugation (12000 x g) for 5 min at room temperature. The extraction efficiency was calculated by:

Brain extraction efficiency = counts per minute (CPM) in supernatant (brain extract)/CPM in brain homogenate extracted

To identify the parent-retention time based on the ultraviolet (UV) chromatogram, 20 µL of a solution containing UCB2713 was added to the supernatant (350 µL), followed by 350 µL of 50 mM ammonium formate (adjusted to pH 8), then 0.5 mL of the resulting solution was injected onto the high-performance liquid chromatography (HPLC) column. Two aliquots of this solution were counted to determine the HPLC recovery as follows:

$$\text{Recovery efficiency \%} = \frac{\text{CPM}_{\text{recovered}}}{\text{CPM}_{\text{expected}}} = \frac{\sum_{i=1}^{30} CPM\ \text{f}_{\text{i}}}{\frac{\text{CPM}_{\text{(aliq.)}}}{Valiq.} * 500\ \mu L} \cdot 100$$

Where "$CPM_{\text{(aliq.)}}$" is the radioactivity measured for the HPLC brain homogenate samples aliquot, "*V*aliq." Is the volume of each aliquot, "CPM f_{i}" is the count per minute for fraction "i" and $\sum_{i=1}^{30}$ is the sum of 30 fractions.

Plasma Sample Analysis

Mouse blood samples were centrifuged at 7500 x g for 3 min at 4 °C and plasma was extracted with a ratio of 1:1 (plasma:acetonitrile). The plasma extraction efficiency was calculated by:

Plasma extraction efficiency = CPM in supernatant (plasma extract)/CPM in plasma extracted

Samples were prepared as described above for the brain, and two aliquots of solution were counted to determine the HPLC column recovery.

HPLC Procedure and Data Analysis

The HPLC system used a pump, degasser, UV-Vis/Diode Array Detector, and fraction collector. HPLC conditions used were 55% acetonitrile and 45% 100 mM ammonium formate (Method 1), or 48% acetonitrile and 52% 100 mM ammonium formate (adjusted to pH 8; Method 2) as the mobile phase at 5 mL/min and an Agilent XDB-C18 semi-preparative

column (5 micron, 250 x 9.4 mm; Method 2); 30 sample fractions (Method 1) and 36 sample fractions (Method 2) were collected over either a 10- or 12-min run.

A radio-chromatogram was reconstructed from the HPLC fractions collected and the percent of unchanged [^{11}C]UCB2713 was calculated:

$$[^{11}\text{C}]\text{UCB2713 parent fraction} = \frac{\sum_{i=n}^{m} cCPM\ \text{f}_{\text{i}}}{\sum_{i=1}^{30} cCPM\ \text{f}_{\text{i}}}$$

Where "$cCPM$ f_{i}" is the radioactivity measured for the HPLC fraction, corrected for the background radioactivity. "n" is first and "m" the last HPLC fraction containing the parent compound ($1 \leq n, m \leq 30$ or 36 [where 30 or 36 are the total number of fractions collected]).

PET-Computed Tomography (CT) Scanning and Image Reconstruction

Three mice were euthanized at the 90 min time point following radioligand injection. Prior to euthanization, these mice were scanned by PET-CT. A dynamic PET scan (Siemens Inveon DPET with docked multi-modality CT scanner) was acquired for each anesthetized mouse following IV administration of [^{11}C]UCB2713. PET images were acquired in list mode and reconstructed with increasing time frames over the duration of the scan to characterize radiotracer kinetics. The Fourier-Rebinning algorithm was applied, and each image

reconstructed using a 2D filtered back-projection algorithm with a ramp filter; the images generated were on a 128 x 128-pixel matrix.

PET-CT image data analysis

Image processing and data analysis at Invicro (London, UK) were performed by a semi-automatic computational pipeline developed in-house using MATLAB™ R2014b. Native PET format images were converted into Neuroimaging Informatics Technology Initiative format and were quality checked for any misalignment between PET and CT. The dynamic PET images were manually re-aligned to CT images if any misalignment was observed. Whole brain regions of interest (ROI) were defined using summed PET images (early and late stages of [^{11}C]UCB2713 uptake) and CT images. Whole brain time activity curves (TAC) were generated from the dynamic PET images using the whole brain ROI.

Radioactivity measurements for blood, plasma, and *ex vivo* brain were decay corrected to the time of radioligand injection and expressed as standardized uptake values (SUV) in units of g/mL:

$$SUV = \frac{Activity}{ID/BW}$$

Where "Activity" is the radioactivity concentration (kBq/mL), "ID" is the injected dose of radioactivity (kBq), and "BW" is the subject body weight (g).

Phase 1 PET Study in Healthy Male Participants (TM0017)

PET-CT Image Analysis

The TAC and *total* plasma data were fitted to a single compartment model to determine the influx and efflux constants K_1 and k_2. Because the plasma free fraction estimate is imprecise, we elected to fit the model using the total (free and bound) arterial concentration input function. The parameters of this model are related to the analogous parameters which would be estimated using an accurate estimate of the free arterial concentration using the equations below. We were able therefore to determine and present the K_1 and estimated whole brain total distribution volume (V_T), with relatively good repeatability between patients, and the equations below could also be used to relate the overall mean estimates back to the ideal total brain to free plasma minzasolmin (V'_T) and unidirectional extraction E_u which can be calculated using the free fraction. The blood volume in the model was fixed at 5%. In this model, radiotracer which is free in tissue, non-specifically bound and specifically bound (i.e. to a target of interest) are lumped together in a single compartment, and so interchange between those states is assumed to be rapid.

The arterial input function used represents the total concentration of parent radiotracer in plasma, with no correction for plasma protein binding and the fitted and observed K_1 from this model is lower than the rate of uptake from the *free* plasma concentration (K'_1):

$$\boldsymbol{K'_1 = \frac{K_1}{f_p^u}} \quad \textbf{Equation 1}$$

K'_1 and hence K_1 are related to rCBF (*F)* and E_u (Morris et al., 2004):

$$\boldsymbol{K'_1 = FE_u} \quad \textbf{Equation 2}$$

To calculate the E_u from the observed K_1 from the model we then need to combine Equation 1 and 2:

$$E_u = \frac{K_1}{F f_p^u} \quad \textbf{Equation 3}$$

The partition coefficient between total brain and total plasma concentration was determined from the fitted K_1 and k_2 parameters:

$$V_T = \frac{K_1}{k_2} \quad \textbf{Equation 4}$$

and the ratio of total concentration in brain to free concentration in plasma (V'_T) could be estimated:

$$V'_T = \frac{V_T}{f_p^u} \quad \textbf{Equation 5}$$

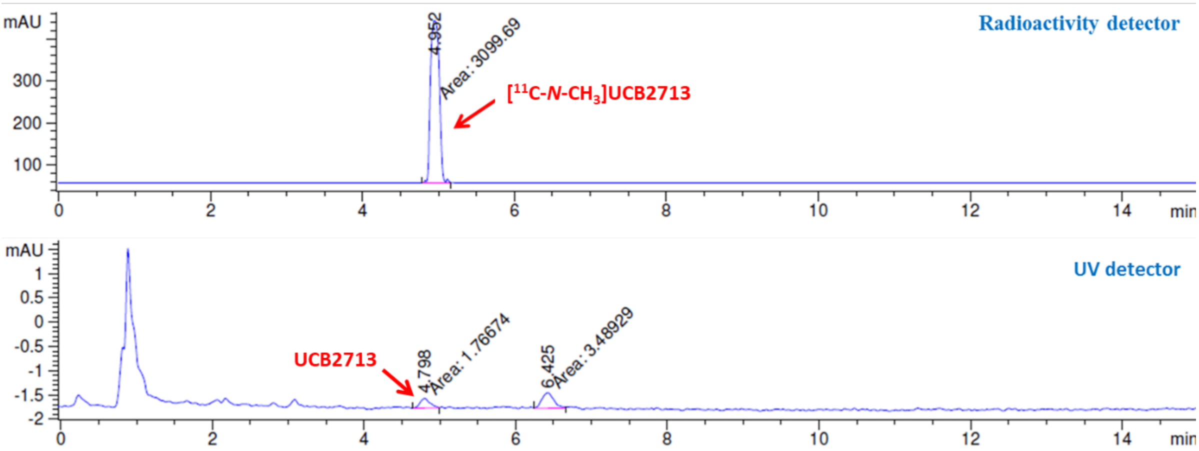

Supplemental Fig. 1. Example of HPLC chromatograms (radioactive trace, top; UV trace, bottom) obtained during HPLC analysis of [^{11}C-*N*-CH_3]UCB2713 dose. The time delay

between radioactive and UV peaks is due to the connection in series of the two detectors, with the UV detector being first.

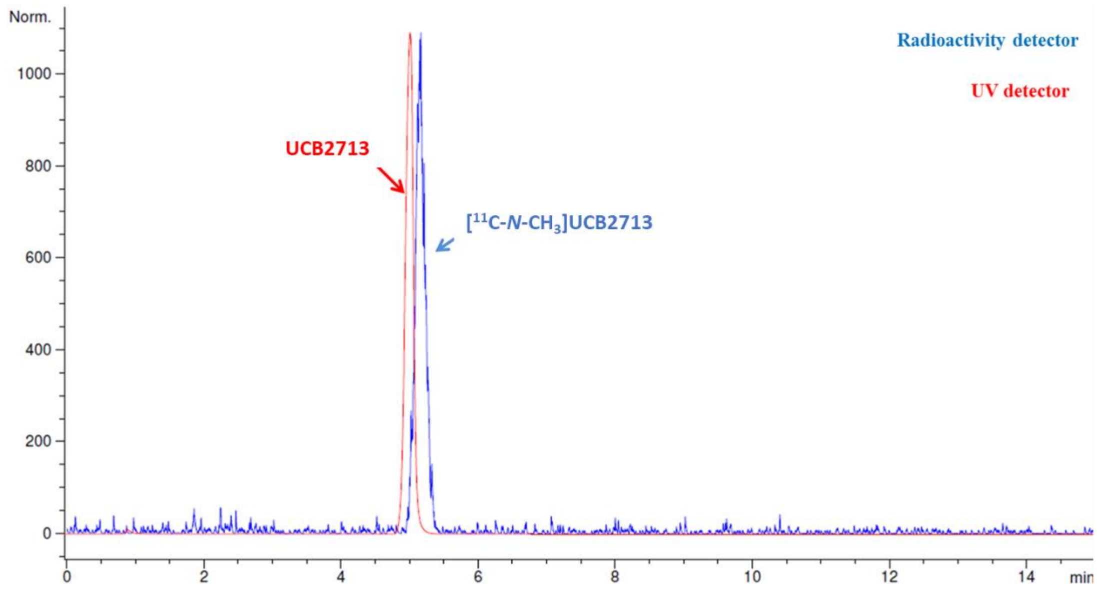

Supplemental Fig. 2. Radioactive and UV HPLC chromatograms overlay obtained during the HPLC analysis of [^{11}C-*N*-CH_3]UCB2713 dose spiked with non-radioactive UCB2713 reference standard. The time delay between radioactive and UV peaks is due to the connection in series of the two detectors, with the UV detector being first.

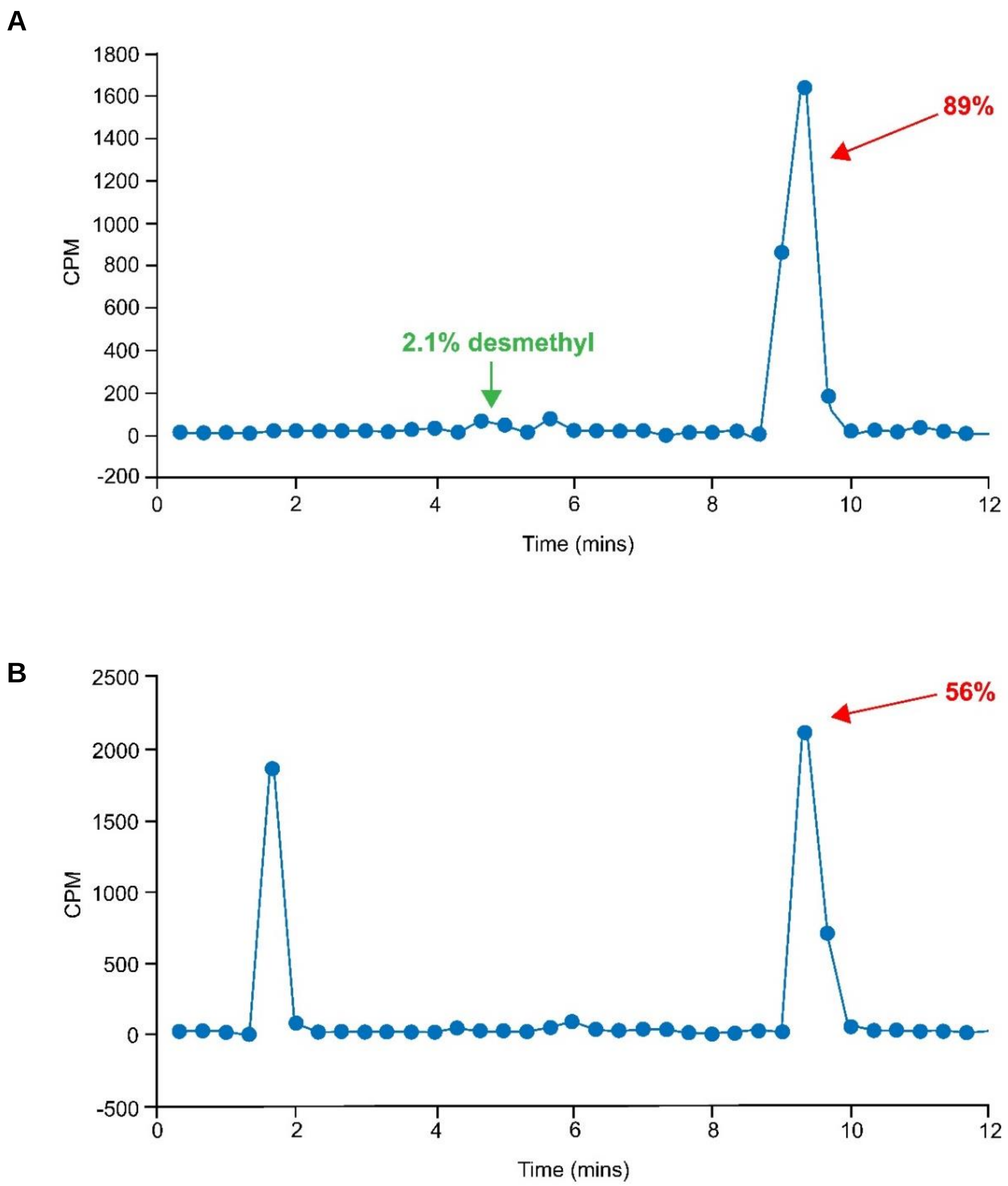

Supplemental Fig. 3. Radio metabolites present in a randomly selected mouse brain for the [11C-CO]UCB2713, 7–20 min **(a)** and [11C-*N*-CH_3]UCB2713, 4–40 min **(b)** tracer.

[11C-CO]UCB2713 [11C]carbonyl UCB2713, *[11C-N-CH_3]UCB2713* [11C]methylamine UCB2713, *min* minute, *CPM* counts per minute

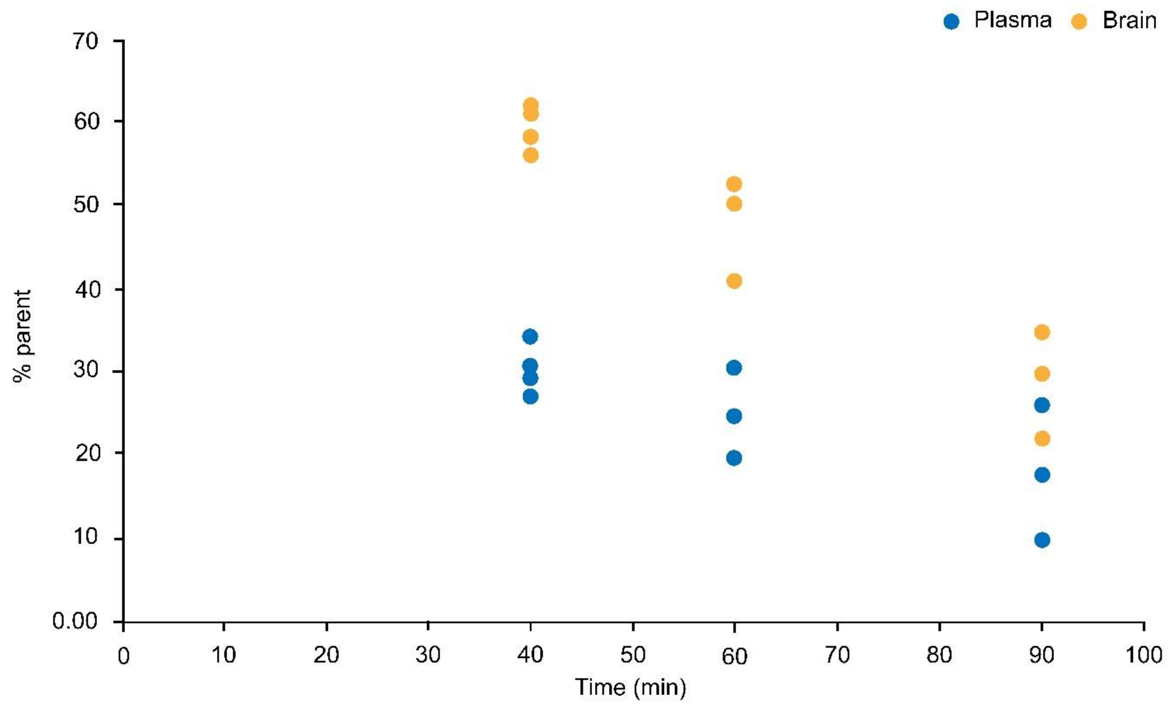

Supplemental Fig. 4. *In vivo* metabolism of [^{11}C]UCB2713 in mouse plasma and brain at specified time points. Data shown are from n = 3 mice. Sampling from one mouse was undertaken at the 40-min timepoint, to confirm suitability of the methodology (originally developed using rat samples) to analyze mouse samples. This initial result demonstrated that the method was satisfactory; the corresponding datapoint is included in the figure for completeness.

min minute

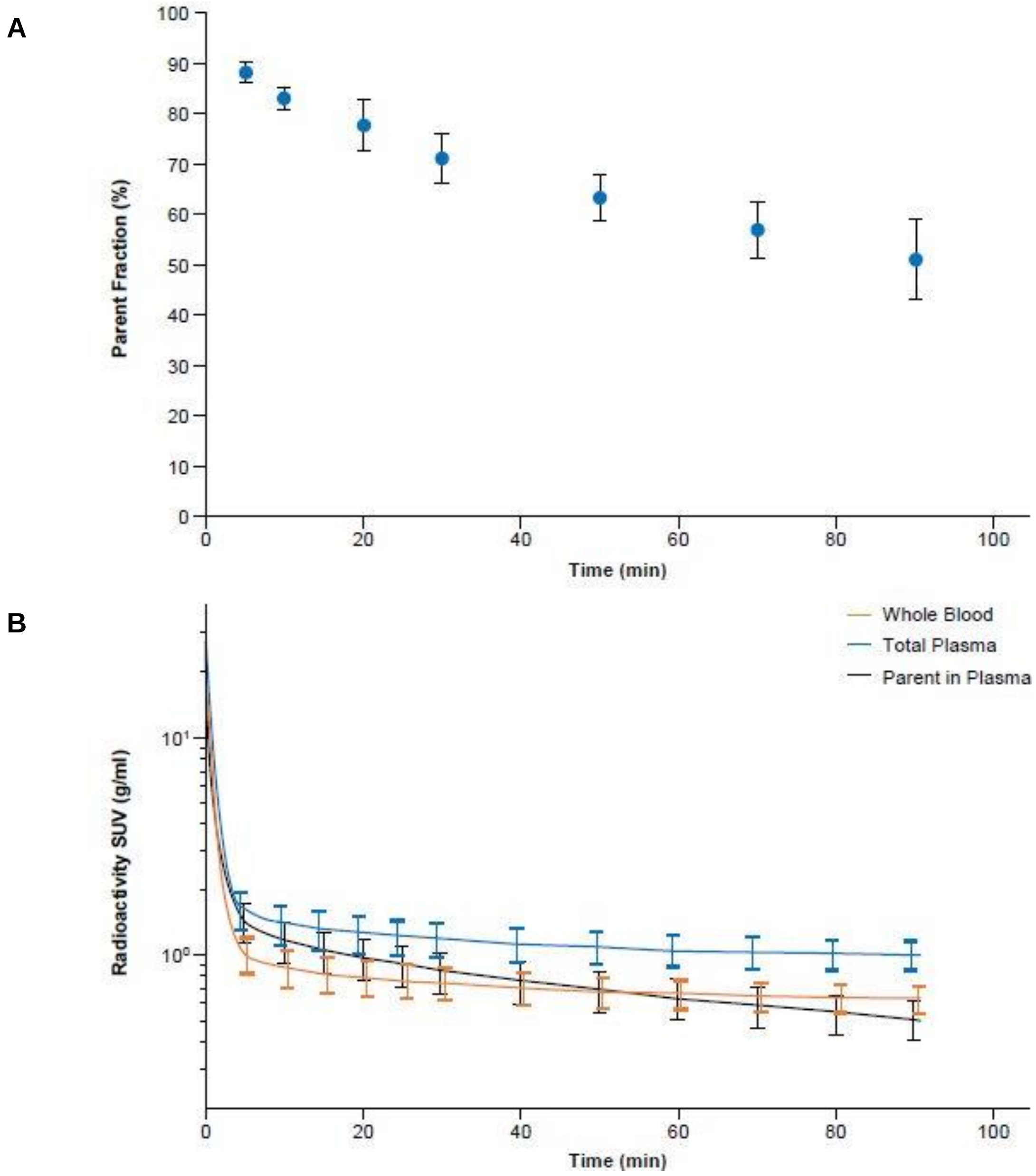

Supplemental Fig. 5. Summary of arterial blood data from eight PET scans of healthy participants (*N* = *4*) from the TM0017 study. (a) Measured metabolite data, plotted as mean values with standard deviation error bars. (b) Input function data derived from continuous and discrete arterial blood samples. Error bars show standard deviations at time points approximately equal to the discrete blood sampling times (offset for clarity).

min minute, *SUV* standard uptake volume
